# Temperature Stress Mediates Decanalization and Dominance of Gene Expression in *Drosophila melanogaster*

**DOI:** 10.1371/journal.pgen.1004883

**Published:** 2015-02-26

**Authors:** Jun Chen, Viola Nolte, Christian Schlötterer

**Affiliations:** Institut für Populationsgenetik, Vienna, Austria; Georgia Institute of Technology, UNITED STATES

## Abstract

The regulatory architecture of gene expression remains an area of active research. Here, we studied how the interplay of genetic and environmental variation affects gene expression by exposing *Drosophila melanogaster* strains to four different developmental temperatures. At 18°C we observed almost complete canalization with only very few allelic effects on gene expression. In contrast, at the two temperature extremes, 13°C and 29°C a large number of allelic differences in gene expression were detected due to both *cis*- and *trans*-regulatory effects. Allelic differences in gene expression were mainly dominant, but for up to 62% of the genes the dominance swapped between 13 and 29°C. Our results are consistent with stabilizing selection causing buffering of allelic expression variation in non-stressful environments. We propose that decanalization of gene expression in stressful environments is not only central to adaptation, but may also contribute to genetic disorders in human populations.

## Author Summary

Gene expression is the most direct link between the genetically encoded information and the phenotype. We analyzed the patterns of gene expression in two different *D. melanogaster* genotypes and their offspring at four different temperatures to determine if gene expression regulation is modulated by temperature. Interestingly, we find that at the intermediate temperature (18°C) alleles from both genotypes have very similar gene expression, suggesting a strong canalization of gene expression despite substantial genetic differences. More extreme temperatures break this canalization and result in many differently expressed genes, caused mainly by *trans*-acting factors. Most of the expression differences are non-additive, with a swap in dominance between the two extreme temperatures.

## Introduction

Canalization, the buffering of a phenotype against environmental or genetic perturbation, has been independently suggested by Schmalhausen [1] and Waddington [2]. Most models of canalization assume that canalization evolves in a response to stabilizing selection (reviewed in [3,4]), but using simulations Siegal and Bergman (2002) found that canalization can also be achieved by regulatory networks even in absence of stabilizing selection [3]. Conversely, genetic or environmental perturbations can result in the loss of canalization (“decanalization”) and thus the release of previously hidden or “cryptic” genetic variation. The perhaps best example for such decanalization induced by strong genetic perturbations comes from a series of experiments focusing on impaired function of the chaperone HSP90 [5–7], but other examples have been described in Drosophila [8–15].

Phenotypes are determined by spatial and temporal patterns of gene expression and environmental and genetic variation has been documented to affect gene expression. How the interplay of genetic and environmental variation affects gene expression can be studied with sequencing based expression profiling (RNA-Seq). Many studies have used expression profiling to study genetically diverged individuals/populations at two environments (e.g.:[16–20]), but the canalization of gene expression across the entire transcriptome still remains understudied. Allele specific gene expression analysis in two inbred lines and their progeny is a popular approach to gain insight into the regulatory architecture of gene expression [21–24]. Here, we expose two *D. melanogaster* strains to four different temperatures to determine how temperature stress affects gene expression. Specifically, we analyze the canalization of gene expression of two genotypes. Furthermore, we expand the analysis of canalization by dissecting the *cis*-and *trans*-effects at different temperatures.

## Results

**Fig 1:**
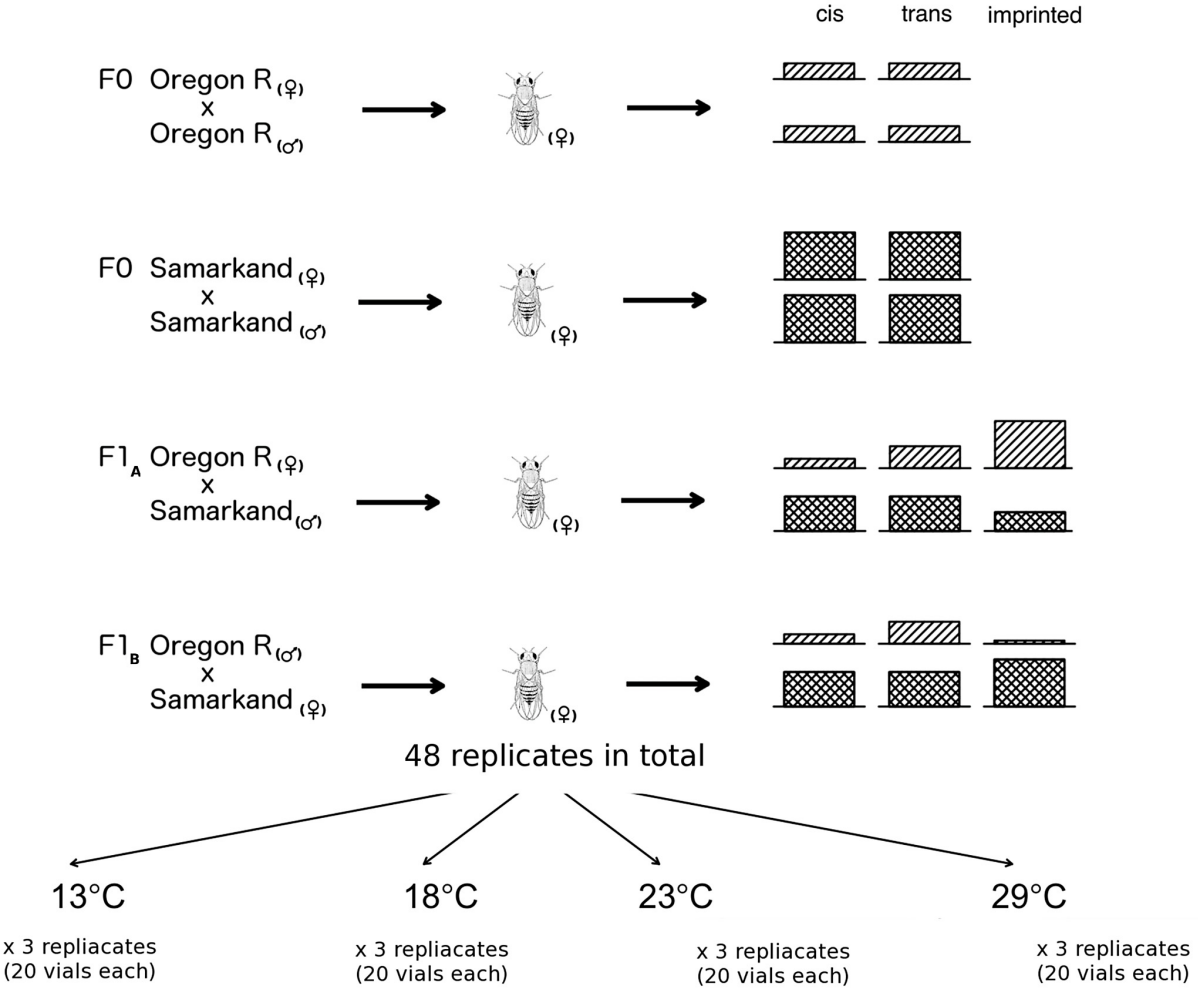
Overview of allele specific expression profiling by RNA-Seq in this study. RNA-Seq libraries were prepared from adult females of four different *D. melanogaster* crosses: two parental inbreds Oregon R and Samarkand, and two offspring crosses in both parent-of-origins (F1_A_ and F1_B_). Three replicates for each cross was reared in four different developmental temperatures and in total 48 replicates were collected in this study.

The two inbred *D. melanogaster* strains Oregon R (O) and Samarkand (S) were crossed and eggs laid at 23°C were transferred to four different development temperatures (13°C, 18°C, 23°C and 29°C, Fig. 1). We measured gene expression by performing RNA-Seq on three replicates of each of the four genotypes in adult females. Paired-end reads were mapped to reference genomes that were modified to avoid preferential mapping of reads from one of the two parental genomes (see also Material and Methods). Since we were interested in allelic differences in gene expression, only those reads were further processed, which mapped unambiguously to one of the two modified reference genomes (see Table 1 and Material and Methods).

**Table 1:** Summary of allele specific mapping.

	Temperature	13°C	18°C	23°C	29°C
**Oregon R**	#raw reads	35,439,037	38,040,969	37,245,502	42,845,478
	#reads mapped^a^	7,029,863	8,619,841	8,291,619	10,621,740
	#reads mapped to features^b^	6,469,806	8,072,438	7,791,375	9,985,236
**Samarkand**	#raw reads	34,443,487	39,811,213	38,976,587	50,131,346
	#reads mapped	6,180,263	7,409,968	8,734,436	12,121,409
	#reads mapped to features	5,553,249	6,705,893	8,115,453	11,295,071
**F1**_**A**_	#raw reads	43,672,116	40,889,194	41,640,367	45,117,839
	#reads mapped	7,612,730	8,117,870	8,673,045	10,400,050
	#reads mapped to oregonR (%)^c^	51.4	51.7	51.3	51.2
	#reads mapped to features	6,945,409	7,483,157	8,063,002	9,661,667
**F1**_**B**_	#raw reads	42,796,478	42,474,298	42,725,914	43,042,101
	#reads mapped	7,721,481	8,532,061	9,036,195	10,166,985
	#reads mapped to oregonR (%)^c^	48.4	47.9	48.6	49.2
	#reads mapped to features	7,036,089	7,784,495	8,381,386	9,471,969

Mean number of read pairs mapped across three replicates are given for in parents and F1 at four temperatures.

a: number of read pairs unambiguously mapped to concordant parental genome

b: number of read pairs mapped to unambiguous regions of *D. melanogaster* annotation feature

c: We noticed a slight imbalance between F1_A_ and F1_B_ for the number of reads mapped to the Oregon R reference. This imbalance is due to mitochondrial gene expression, which approximates about 1% of all reads. Since, F1_A_ has the mtDNA genome from Oregon R, more reads map to the Oregon R reference.

### Almost complete absence of imprinting in Drosophila

Comparing the F1 individuals from crosses of Samarkand with Oregon R in both directions we tested for the presence of imprinting in our experiments. Consistent with previous results [25–27], levels of genes expression were very similar between the two genotypes, indicating the absence of imprinting in *D. melanogaster*. Only two genes, chrU_5299041_5299681.0 and CG1275, showed significant parent-of-origin effect (FDR = 5.6e-19 and 0.02, S4 Fig.). While the expression difference of CG1275 was small, chrU_5299041_5299681.0 encoding the cytochrome b gene differed more than 5.2e4-fold between F1_A_ and F1_B_. This result indicates a different mitochondrial gene expression rather than imprinting. After excluding both genes, we combined the data from F1 offspring from crosses in both directions.

### Decanalization of gene expression between parents

**Fig 2:**
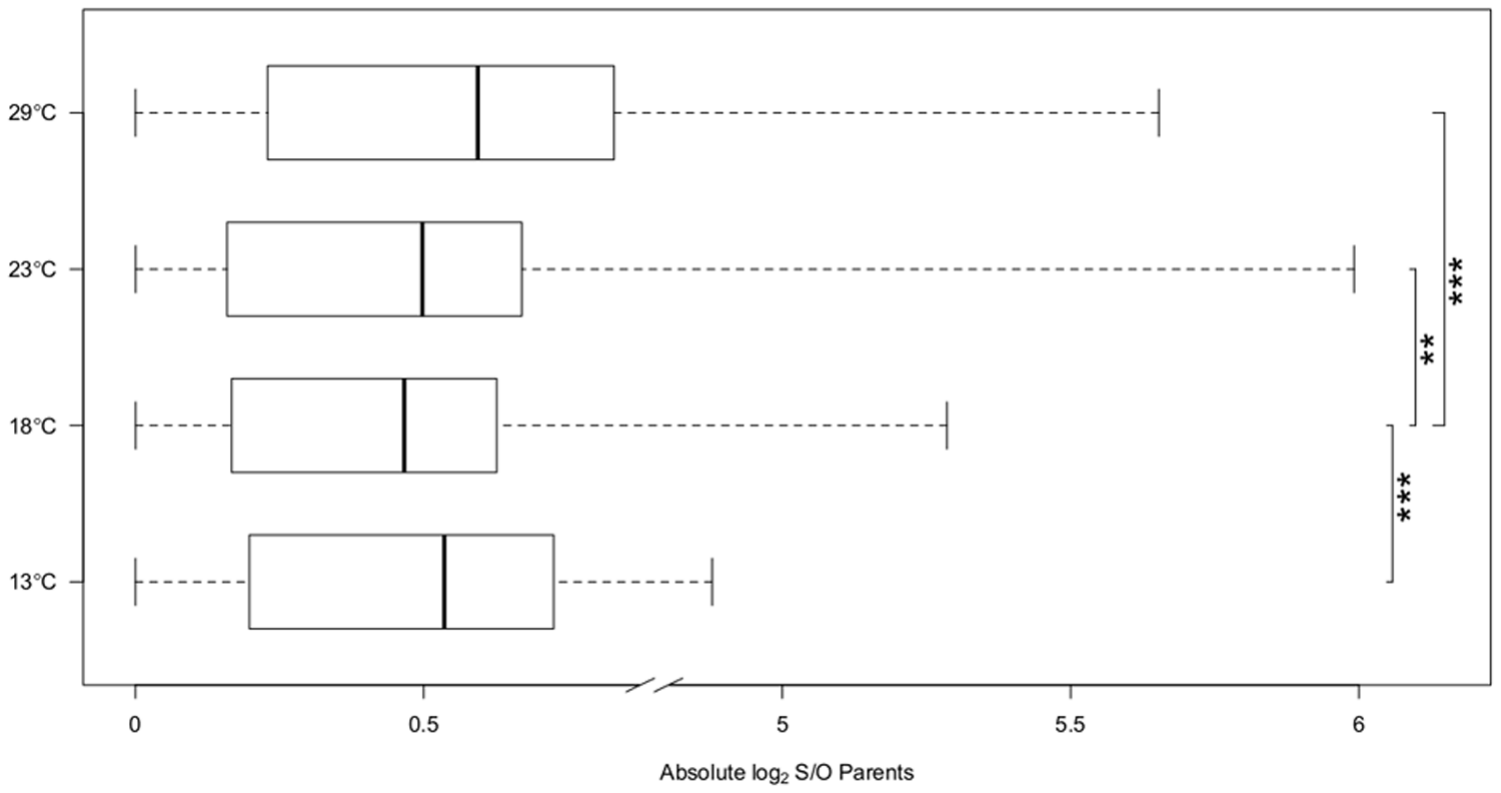
Expression divergence between Oregon R (O) and Samarkand (S). The box plots of the absolute log_2_ expression ratio in Samarkand and Oregon R parents across different temperatures. At 18°C the expression pattern between the two strains is most similar, while at the most extreme temperatures the expression is most diverged, suggesting temperature associated decanalization of gene expression. *P*-values are present for pairwise comparisons between 18°C and three other temperatures (‘**’<0.01, ‘***’ <0.001).

7,189 genes were expressed in parents and offspring and showed allelic differences between the parental strains Oregon R and Samarkand. Comparing the gene expression profiles between the two parental strains we observed a striking difference in gene expression divergence for flies grown at different temperatures (13°C, 18°C, 23°C and 29°C) (Table 2). At 18°C only 92 genes (1.2%) differed significantly in gene expression between Oregon R and Samarkand. In contrast, for flies either kept at 13°C or 23°C the number of differentially expressed genes increased to more than 1,000 (15%). The largest difference between the parental strains was observed at 29°C, where 2,581 genes (32.9%) were differentially expressed. The same trend of canalization/decanalization was seen when we compared the absolute expression difference between Oregon R and Samarkand strains across all expressed genes, with flies at 18°C exhibiting the lowest absolute log_2_ fold-difference (mean = 0.47, Fig. 2), followed by flies at 23°C (mean = 0.50, Fig. 2). Flies maintained at 13°C (mean = 0.54, Fig. 2) and 29°C (mean = 0.59, Fig. 2) had the highest difference in gene expression (all pairwise differences were highly significant; Wilcoxon test, FDR<0.01,).

**Table 2:** Summary of genes with expression differences.

		**13°C**	**18°C**	**23°C**	**29°C**
**Divergence in F0** **Allelic expression divergence**		1016	92	1210	2581
	Ambiguous (ambig)	2375	1246	1248	1789
	Not different (n.s.)	3717	5855	4911	3369
	Compensatory	281	18	86	104
	*cis* × *trans*	18	0	13	31
	*cis* + *trans*	144	15	75	77
	*trans* only	501	17	485	1586
	*cis* only	153	38	371	233
**Inheritance modes of gene expression**	Not different (n.s.)	3470	7162	6375	4878
	O-dominant (O-dom)	153	11	200	2230
	S-dominant (S-dom)	3287	16	553	52
	Additive	71	0	48	17
	Over- dominant	85	0	13	7
	Under-dominant	123	0	0	5

To eliminate a potential bias in gene expression differences caused by variation in sequencing depth, we down-sampled the data sets to similar read counts by randomly picking paired-end reads without replacement. The final number of reads was determined ranking for each temperature the replicates by the number of reads. For each rank the number of reads was matched to the one with the smallest number of reads. This procedure maximized the number of reads to be included in the matched samples (S1 Table). Importantly, we found the same trends in the down sampled data (S2 Table). Additionally, to rule out that heterogeneous read coverage across the whole gene body could affect our results, we summarized the gene expression differences using only reads aligned to the 500bp at the 3’end. Again, the same trends remain (S3 Table).

Since we detected in three libraries (t29-rep2 OregonR, t23-rep2 Samarkand, and t18-rep3 F1A) expression of male specific genes we suspected that a small fraction of males were inadvertently included in the flies used for RNA extraction. In order to rule out that any of the conclusions drawn in this manuscript we removed the contaminated samples as well as two other libraries of similar sizes to balance the replicate size in that temperature (see S1 Table). In addition, we used only reads mapping to the 500bp at the 3’ end. The analysis using this reduced data set we confirmed the same patterns we saw for the full data set, albeit less pronounced due to the reduced power (S4 Table).

### Decanalization of cis- and trans-regulation

**Fig 3:**
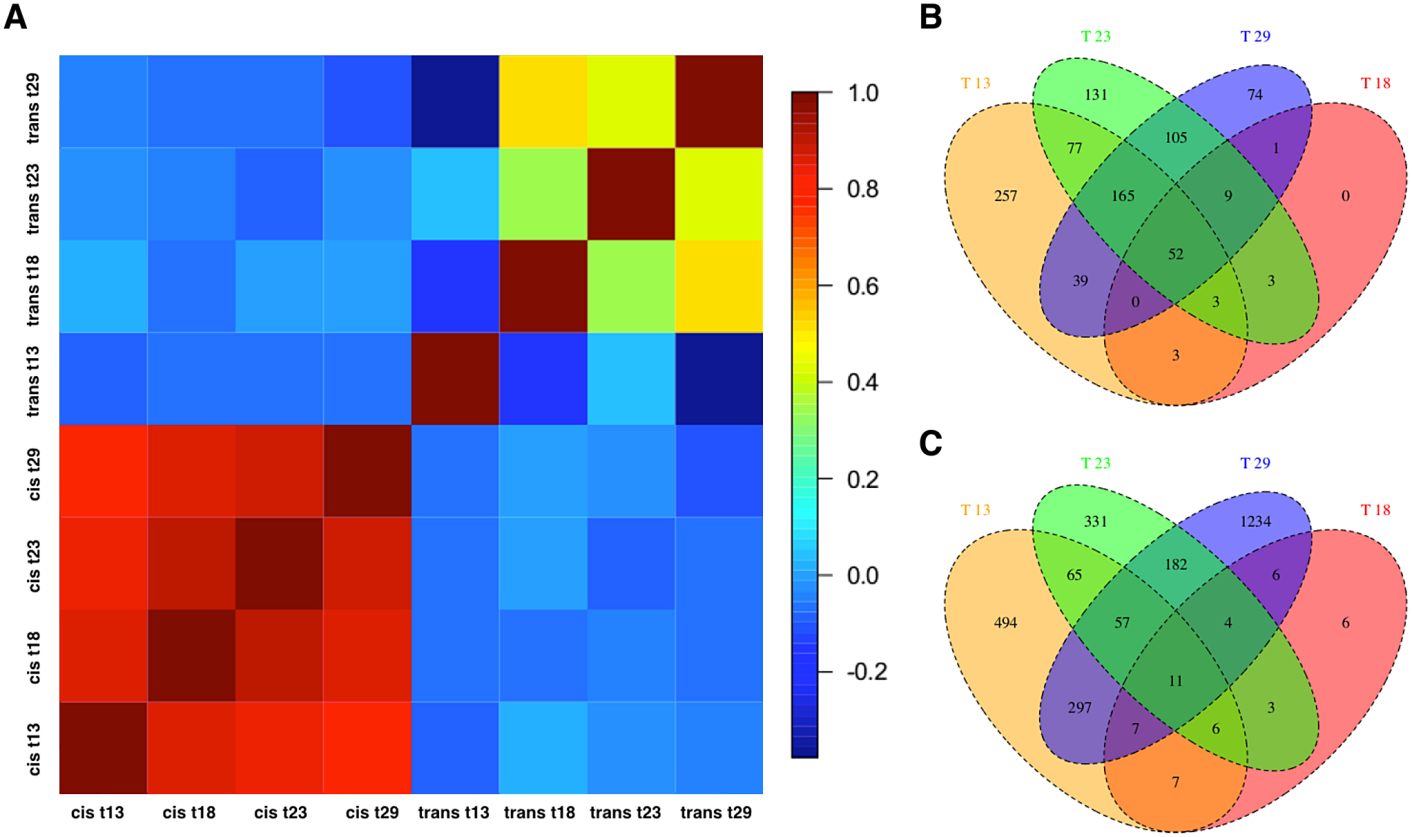
Temperature dependence of *cis*- and *trans*-regulatory differences. Scatter plots contrasting the relative allelic expression levels of parents and F1 offspring. Since the large number of genes makes a quantitative assessment difficult, we also show the density distribution for each class of genes. For representation purposes density distribution of genes with no significant differences in gene expression (yellow) is scaled by 1/10. While at (B) 18°C almost no allelic heterogeneity is present, the number of *cis*- and *trans*-effects increases with more extreme temperatures, (A) 13°C (C) 23°C and (D) 29°C.

**Fig 4:**
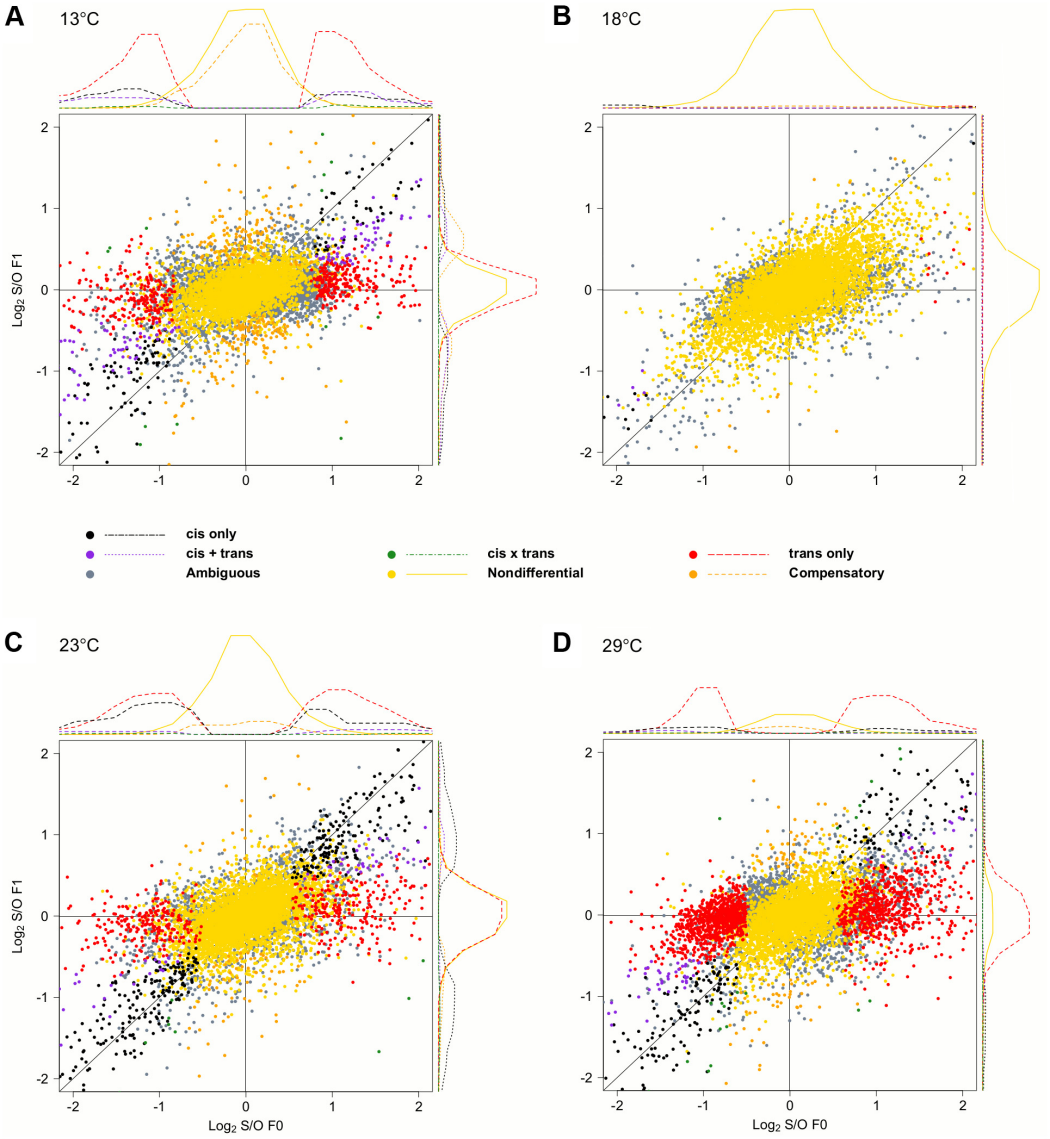
Temperature-dependence of *cis*- and *trans*-effects. (a) Pairwise correlation coefficient matrix (Spearman’s *r*) between *cis*-effects and *trans*-effects across all temperatures. The correlation of *cis*-effects across environments was more similar than the one of trans-effects. (b) Venn Diagram showing the number of *cis*-regulated and (c) *trans*-regulated genes at four different temperatures.

Next, to dissect the regulatory architecture underlying these temperature specific effects, we measured allele specific gene expression by comparing expression of Oregon R and Samarkand alleles in both parents and offspring (Fig. 3). At 18°C, only a small fraction of genes had significant *trans*-regulatory (50, 0.7%) and *cis*-regulatory (71, 1.0%) effects. Flies maintained at 23°C, had approximately 1,000 genes with allelic regulatory divergence: 659 (9.2%) with *trans*-regulatory effects and 545 (7.6%) with *cis*-regulatory effects. For the two most extreme temperatures 944 (13.1%) genes differed in allelic expression due to *trans*-acting variants at 13°C and 1,798 (25.0%) at 29°C. The number of genes affected by *cis*-regulatory variants was 596 (8.3%) at 13°C, and 445 (6.19%) at 29°C (Table 2). Importantly, these results were not affected by different library sizes, since we obtained similar results for a down sampled data set (S2–S3 Tables). Thus, consistent with previous results [28] we found that temperature stress affects both *cis*- and *trans*-regulatory variants, with *trans*-effects being more common. When we compared the effect sizes by correlating *cis*- and *trans*-effects across temperatures, we found a striking difference. While *cis*-effects changed moderately with temperature difference (mean Spearman’s *r* = 0.86, Fig. 4a), *trans*-effects were practically uncorrelated at different environmental temperatures (mean *r* = 0.14, Fig. 4a, c). Previous studies contrasting different yeast strains have identified a similar pattern of pronounced *trans*-effects under stressful conditions [23,29]. In our study *cis*-regulated genes are also condition-dependent. Among the genes with temperature-specific *cis*-effects a considerable fraction did not show any *cis*-effects at any other temperature (13°C: 43%, 23°C: 24%, 29°C: 16%, Fig. 4b).

Until now, we analyzed each temperature separately, hence we complemented this analysis estimating allelic difference, temperature, and their interaction jointly. Consistent with our other analysis this approach identified at least 1200 genes differing in expression level between F0 strains across four temperatures (S4 Table). We also identified 480 genes were regulated by *cis*-effect across all temperatures while the number of trans-effects was close to 4,994. We found that 22 *cis*-effects and 4,300 *trans*-effects had significant interactions with temperature. This confirms that both regulatory effects are condition-dependent, and that *trans*-effects are more pronounced. For about 5,000 genes the expression level changed significantly across temperatures in either F0 or F1 datasets. 3,336 of them were found in common as temperature response genes in all tests (a detail gene list can be found in S1 Dataset).

### Dominance of gene expression

**Fig 5:**
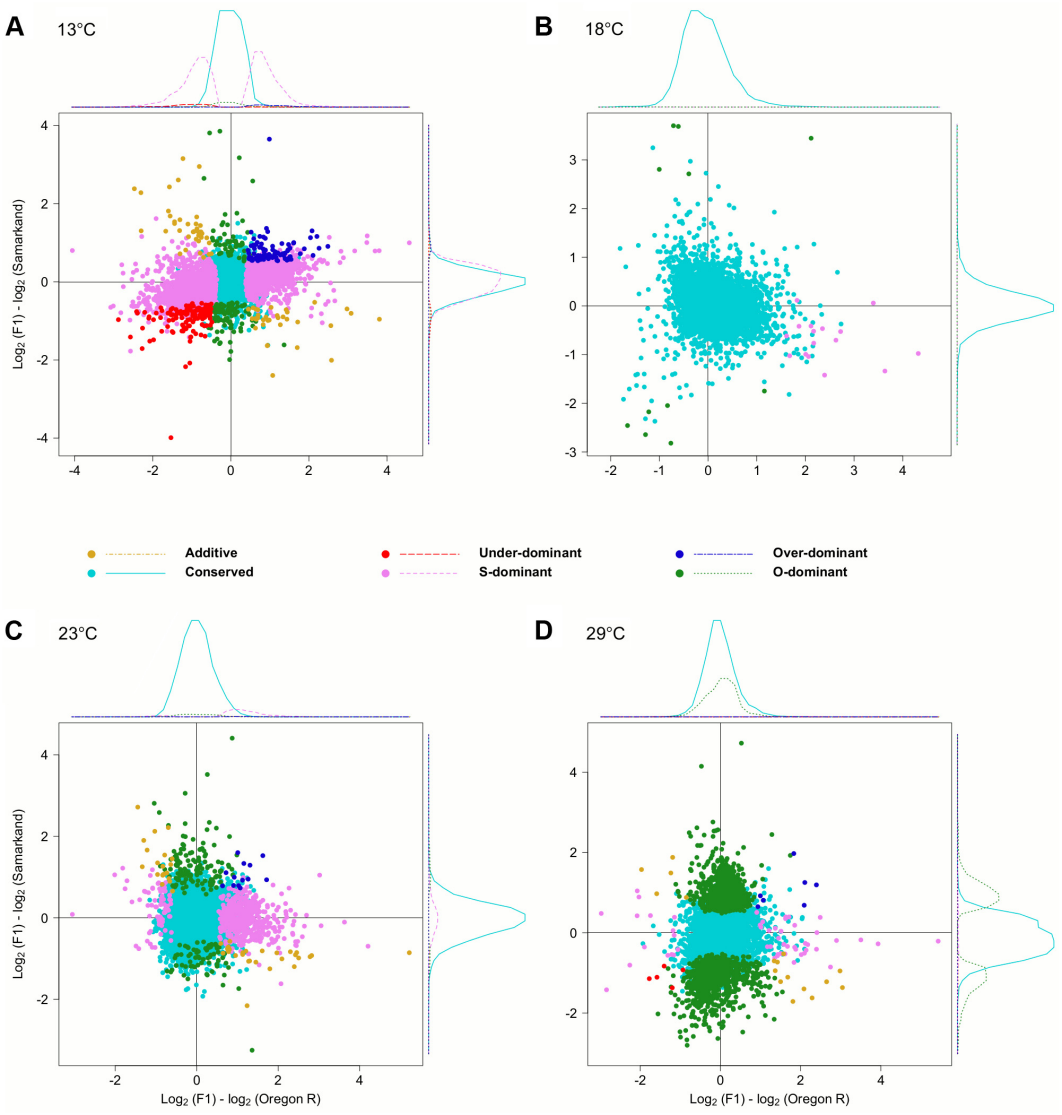
Temperature dependent inheritances of gene expression levels. Scatter plots summarizing the inheritance patterns by contrasting the differences of each of the parental lines to the F1. Since the large number of genes makes a quantitative assessment difficult, we also show the density distribution for each class of genes. . For representation purposes density distribution of genes with no significant differences in gene expression (light blue) is scaled by 1/10. Again, at (B) 18°C the fewest differences were noted compared to (A) 13°C, (C) 23°C, and (D) 29°C.

To test the influence of the large number of *trans*-effects, we determined the mode of inheritance (i.e.: degree of dominance) by comparing the total expression levels of offspring to that of both parents (S3 Fig.). Most genes showed either no difference or were dominant, and only few genes (<3.9%) were classified as being additive, under-dominant, and over-dominant (Fig. 5). Again, we noticed a very striking dependence on temperature, with flies at 18°C showing the least differences between parents and offspring. Only 27 genes showed dominance in gene expression. At 23°C, 200 genes (2.8%) were Oregon R-dominant, and 553 (7.7%) were Samarkand-dominant. At the two extreme temperatures, up to 51.7% of the expressed genes showed dominance, but the distribution was not balanced between the two strains (Table 2). While at 13°C the majority of dominant alleles (3,287, 45.7%) had an expression level resembled the Samarkand parent with non-significant or negligible change (<1.25-fold), at 29°C the majority (2,230, 31%) resembled the Oregon R parent. Among the genes with dominant gene expression, only a moderate number showed allelic imbalance: 504 (14.6% of dominant genes) at 13°C, 19 (70.4%) at 18°C, 267 (35.5%) at 23°C, and 244 (10.7%) 29°C, suggesting that for the majority of genes both parental alleles are subject to the same regulatory input.

### “Dominance-swapped” genes are enriched for a few transcription factor binding sites

Among the genes with a dominant expression pattern at either 13°C or 29°C, 1,384 genes were dominant in both environments, but their dominance pattern was reverted between the two temperatures (hereafter we refer to these genes as “dominance-swapped” genes). Since among these swapped genes the fraction of *trans*-regulated ones is very high (up to 66%), we reasoned that they may be regulated by a few transcription factors (TFs). We therefore performed an *in silico* analysis of enrichment for transcription factor binding sites among these dominance-swapped genes. The binding sites of 13 TFs were significantly overrepresented (S5 Table). We further investigated whether a combination of TFs may be enriched, rather than a single one. Indeed, we found that several combinations of TFs may affect the expression of dominance-swapped genes. The most highly ranked combination of *Chro* and *BEAF-32* may affect the expression of up to 934 genes (68% of the dominance swapped genes), while the largest combination contained 11 of the 13 TFs with binding site enrichment in the single TF analysis (S6 Table).

Eight out of these 13 TFs were present in our data set (Table 3). Among these, three TFs showed dominance and two of them also exhibited swapped dominance. Three TFs had *cis*- (*mip120*) or *trans*-effects (*Med*, *BEAF-32*, and *mip120*). Of particular interest is the transcription factor *mip120*, which is affected by *cis*-regulatory variants at 29°C and at 13°C it is regulated by both *trans*- and *cis*-regulatory variants. It is possible that the *cis*-regulatory variants are responsible for *trans*-regulatory divergence of multiple downstream TFs, causing the coordinated expression of hundred of genes (i.e. sensory *trans* factors *sensu* [23]).

**Table 3:** Allele specific expression and inheritance patterns for transcriptional factors enriched in dominance swapped genes.

	**Genes**	**13°C**		**18°C**		**23°C**		**29°C**	
**Transcription factors**		Allelic	Inherit	Allelic	Inherit	Allelic	Inherit	Allelic	Inherit
	*mip120*	***cistrans***	**S-dom**	n.s.	n.s.	ambig.	n.s.	***cis***	**O-dom**
	*Med*	n.s.	n.s.	n.s.	n.s.	ambig.	n.s.	***trans***	**O-dom**
	*BEAF-32*	n.s.	**S-dom**	n.s.	n.s.	n.s.	n.s.	***trans***	**O-dom**
	*trx*	n.s.	**S-dom**	n.s.	n.s.	ambig.	n.s.	n.s.	n.s.
	*dl*	ambig.	**S-dom**	n.s.	n.s.	n.s.	n.s.	n.s.	n.s.
	*CtBP*	n.s.	n.s.	n.s.	n.s.	ambig.	n.s.	ambig.	n.s.
	*phol*	n.s.	n.s.	n.s.	n.s.	n.s.	n.s.	n.s.	n.s.
	*Chro*	n.s.	n.s.	n.s.	n.s.	n.s.	n.s.	n.s.	n.s.

To shed more light onto the biology of the dominance-swapped genes, we performed Gene Ontology (GO) and pathway analyses and found many genes involved in macromolecule biosynthesis, especially mRNA translation to be overrepresented (S7 Table). Gene ontology categories for cellular components were enriched for mitochondrial ribosome and vitelline membrane. Other molecular functions and bioprocesses had also been identified, annotated as neural signal transmitting, transmembrane transporter activity, and body fluid secretion. On the other hand, genes with *cis*- and *trans*-regulatory effects are not enriched for any GO categories or pathways (after removing genes common with dominance-swapped genes).

## Discussion

Analyzing allelic effects on gene expression for flies grown at four different developmental temperatures we found the striking pattern of a large number of *trans*-effects at stressful temperatures. Similar results were obtained for yeast where stressful culture conditions resulted in large *trans*-effects [23,29] and *C*. *elegans* where 59% of the *trans*-acting genes showed a significant eQTL by environment interaction [28]. The important difference of our study to the previous ones is that we tested multiple temperatures covering stressful and less stressful conditions. Since for flies grown at 18°C the genetic differences between the two strains had almost no influence on the levels of gene expression, our data provide experimental evidence for canalization of gene expression. At stressful conditions, this canalized gene expression is disturbed resulting in many significant differences between the two parental strains as well as significant *cis*-and *trans*-effects.

Following the widely accepted hypothesis that canalization of traits is the consequence of stabilizing selection (reviewed in [3,4]), our data suggest that the most benign temperature for *D. melanogaster* is 18°C. A similar line of argument has as been applied to the evolution of reaction norms. For fitness related traits, such as egg production, it has been proposed that the maximum egg production should be observed for the optimal temperature. While our experiment suggested an optimal temperature of 18°C, the optimal temperature for ovariole number [30] and fecundity [31] is closer to 23°C than to 18°C. It is thus not clear if the reaction norm of fitness traits could serve as good indicator for optimal temperatures. On the one hand the reaction norms tend to be very similar for flies collected from different environments [30,31], while on the other hand the optimal temperature seems to differ among reaction norms. Finally, thermotactic studies demonstrated that 18°C is the preferred temperature of *D. melanogaster* [32,33], suggesting that flies prefer temperatures with canalized gene expression.

Our study has relied on two old laboratory strains that have been established more than 80 years ago [34], and since that time they have probably largely been maintained at 18°C to reduce the number of transfers. We can therefore not rule out that canalization might have evolved to match these common laboratory culture conditions. Nevertheless, comparison of various *D. melanogaster* strains at 25°C identified several hundred differentially expressed genes [35]. Similarly, a study based on the DSPR lines detected 7922 eQTLs at 25°C [36], demonstrating considerable allelic effects in gene expression. While a proper comparison would require an analysis of gene expression at different temperatures, the substantial number of significant differences in gene expression among genotypes at 25°C suggests that gene expression was also decanalized in these studies. Thus, our observation of strongly canalized gene expression at 18°C may not be specific to the strains used in our study.

By uncovering otherwise hidden genetic variation decanalization could facilitate adaptation of populations exposed to a novel, stressful environment. On the other hand, many of the otherwise cryptic variants are expected to be deleterious as recently pointed out by Gibson et al. [37]: environmental and/or genetic stress associated with the recent history of human populations might have resulted in decanalization and a consequence in an increase of complex human genetic disorders. General stressors, such as smoking, have been shown to be a contributing factor to many complex diseases [38]. While it is not proven that decanalized gene expression is causative for these diseases, it is remarkable that for some genetic disorders allelic imbalance in gene expression has been observed [39–42]. Our results considerably strengthen the notion that decanalization might commonly manifest itself as a major allelic imbalance in gene expression.

Like most studies on canalization [5,43,44], our analysis was based on naturally occurring variants. Since, purifying selection is removing deleterious variation and directional selection increases the frequency of favored variants, it may be possible that natural selection could influence our interpretation of canalization. Assuming that regulatory variants are temperature specific and purifying selection is more effective at 18°C, it may be possible that variation influencing gene regulation at 18°C is purged from the population, while the other variants remain segregating in the population. Alternatively, new mutations occurring during the propagation of the classic laboratory strains may have been purged more efficiently at 18°C since laboratory strains are typically maintained at this temperature, albeit at very small population sizes. The outcome of such scenarios would be indistinguishable from the pattern seen in our study. Consistent with the idea of natural selection mimicking the effect of canalization a recent study failed to verify the previously described effect of the histone variant HTZ1 on mutational robustness in mutation accumulation lines for which the effect of natural selection is minimized [45]. Our current understanding of the regulation of gene expression is not sufficiently advanced to decide if sequence variants are affecting gene expression in a temperature specific manner such that selection could remove variants specific to 18°C but other variants could accumulate in natural populations. Future experiments employing mutation accumulation lines or experimental evolution will be instrumental to distinguish between the two scenarios.

The phenomenon of temperature dependent dominance in gene expression seen in our experiments is particularly interesting. A high frequency of genes with dominant modes of inheritance in gene expression has already been documented in other studies in Drosophila [24,46], yeast [47] and *A*. *thaliana* [48]. The novelty of our study is, however, that for a large fraction of genes this mode of inheritance depends on temperature: genes with dominance of the Samarkand allele are showing dominance of the Oregon R allele at the other temperature extreme (Table 2). Our *in silico* analysis suggested that many genes with such a pattern of swapped dominance are potentially regulated by a few transcription factors, of which two also exhibit a temperature dependent swap in the mode of inheritance (Table 3). While it is conceivable that these transcription factors are part of a regulatory network of the swapped genes, the question of the regulation of one or a few master regulators deserves special attention. One simple way to achieve dominance in gene expression is loss of function of one of the parental alleles. This model, however, seems not to apply to our data since the genes are expressed in both parental strains. Alternatively, the dominance may be the result of a different tissue representation in the two parental strains. It has been described that ovary sizes/ovariole number differ among *D. melanogaster* strains [e.g.: 49]. If the F1 does not have an intermediate ovary size but are similar to one of the parents, genes predominantly expressed in the ovaries are expected to show dominance. We tested the hypothesis of differential ovary sizes by comparing the expression of chorion genes and did not find support for this hypothesis since they largely showed no dominance pattern (S8 Table). We also excluded that the swapping dominance is the outcome of a temperature × parental origin interaction (688 swapped dominance genes in F1_A_ and 979 in F1_B_, S9 Table). One interesting observation in *C*. *elegans* linked dominance in gene expression to nonsense mediated mRNA decay (NMD) [50]: in NMD impaired individuals many mutations were highly dominant, while with a functional NMD the same mutations were recessive. While it is conceivable that NMD activity may be affected by temperature, it is not clear how the swapped dominance could be caused. Finally, dominance may be the outcome of some form of allelic crosstalk, either by transvection [51–53] or other mechanisms [54]. How environmental signals are incorporated in such a mechanism, remains an open question.

## Materials and Methods

### Animal rearing and handling

Flies were reared on standard cornmeal-molasse-yeast-agar medium and maintained at 12 h light/12 h dark conditions. Prior to crossing, the parental strains were subjected to 7 generations of sibling pair matings in order to reduce residual heterozygosity. Virgin females of either strain were used for the following four crosses: O female × O male, and S female × S male (F0 parents); O female × S male (hybrid cross F1_A_), and S female × O male (hybrid cross F1_B_). For each type of crosses, three replicates were set up in parallel, each consisting of approximately 80 crosses of a single female and a single male. After two days of egg laying at 23°C four subsets of 20 vials was transferred at four different temperatures (13°C, 18°C, 23°C, and 29°C).

### Sample preparation and sequencing

Virgin females were collected from both parents and F1 flies shortly after eclosion and aged three days before shock-freezing in liquid nitrogen. For each replicate (out of a total of 48), approximate 30 females were homogenized in peqGOLD TriFast Reagent (Peqlab, Erlangen, Germany) using an Ultraturrax T10 (IKA-Werke, Staufen, Germany). Total RNA was extracted, quality-checked on agarose gels, and quantified using the Qubit RNA Assay Kit (Invitrogen, Carlsbad, CA, USA). Paired-end Illumina mRNA libraries were generated from 5μg total RNA. After DNase I treatment (Qiagen, Hilden, Germany), poly(A) transcripts were isolated using the NEBNext Poly(A) mRNA Magnetic Isolation Module (New England Biolabs, Ipswich, MA). Strand-specific paired-end libraries were prepared using the NEBNext Ultra Directional RNA Library Prep Kit for Illumina and size-selected on AMPureXP beads (Beckman Coulter, CA, USA) aiming for fragments between 380–500bp. All libraries were amplified with 12 PCR cycles using index primers from the NEBNext Multiplex Oligos for Illumina Kit (New England Biolabs, Ipswich, MA) and sequenced on a HiSeq2000 using a 2×100bp protocol. Our barcoding scheme was such that all 16 samples of a single replicate were included in the same lane(s) to minimize lane effects.

### Allele specific gene expression

S1 Fig. illustrates the whole allele specific mapping procedure and statistic analyses applied in this study. We first trimmed all sequence reads using the Mott algorithm implemented in PoPoolation [55] and aligned reads of the two parents separately to the Flybase *D. melanogaster* 5.49 assembly. We identified substitutions in the two parental strains relative to the reference genome using variants occurring at a frequency ≥ 0.98 with a read-depth ≥2. Those variants were used to perform a second round SNP-tolerant read alignment using GSNAP [56,57] in order to get the final parental specific SNP datasets with higher confidence. A total number of 177,107 and 193,699 SNPs (i.e.: variants relative to the reference genome) were identified in Oregon R and Samarkand respectively. Amongst those, 182,123 SNPs differed between two parents. The read depths of SNPs were high correlated between two parental data sets (Pearson’s cor. coef = 0.94, *p*-value <2.2e-16, S2 Fig.), suggesting the SNP discovery had suffered marginal bias towards either of parents. We generated two parental specific genomes by modifying the *D. melanogaster* reference using Oregon R and Samarkand SNP datasets.

Reads were assigned to one of the parental genotypes by aligning them simultaneously to both parental genomes. Only unambiguously mapped reads in proper pairs were assigned to either O or S. Reads mapping to both genomes equally well were not included in the analyses. Allelic expression was measured using the ReCOG software tool (https://code.google.com/p/recog/) on each of the two reference genomes separately. We only counted reads that were mapped fully within the gene boundaries. The only exceptions were overlapping genes. Here, the overlapping region was considered ambiguous and not counted. On average 21% of the reads could be assigned to one parental reference genome. Using the RNA-Seq reads from the parents we discovered that 0.19% and 0.27% of the reads were assigned to the wrong parental genome. To account for these potential mapping errors, we followed a previously suggested strategy [58] and simulated about 18,000,000 paired-end reads from both parental genomes and assigned them to either of the reference genomes using the same strategy as for the real data. Equal expression levels for both alleles were expected in the simulated data for all genes, given no mapping errors. Therefore, we excluded 15 genes with simulated allelic expression divergence to avoid biased divergence estimation caused by mapping errors. The protocols described above are implemented in the package ALLIM [58].

Finally we filtered for a minimum expression level using the following criteria. A gene is defined as expressed in the F0 flies if in all samples at least one read was mapped and at least one sample had ≥20 counts. In F1 flies the expression counts to both reference genomes were considered jointly and the same cutoffs as for the parental samples was applied. For a gene to be considered expressed in all samples, the criteria for F0 and F1 flies had to be met. Out of 18,764 *D. melanogaster* genes / features annotated in either Flybase r5.49 [59] or the developmental transcriptome [60], we detected the expression of 7,853 (41.8%) genes in F0 flies, 7,208 (38.4%) genes in F1 flies, and 7,191 (38.3%) genes in both F0 and F1 flies.

### Identifying imprinted genes

We used the F1 crosses that were carried out in both directions to identify imprinted genes. Read counts of each gene were normalized by total library size and RNA composition of each data set using a trimmed mean of M-values method [61]. For each gene, a generalized linear model (GLM) was applied to evaluate the divergence of allelic expression levels between the F1 crosses in two parent-of-origin orders (F1_A_ and F1_B_), at four temperatures separately:

$$log_{2}{\left(\frac{O}{O+S_{}}\right)}_{F1}\sim \left(\begin{array}{l} F1_{A}\\ F1_{B} \end{array}\right)+\varepsilon$$

where ε denotes the error term and the quasi-binominal distribution was used to account for the over-dispersion. *P*-values were calculated by F-test followed by FDR correction. Two putative imprinted genes were excluded from our further analyses of expression divergence, which resulted in a total number of 7,189 genes expressed in F0 and F1.

### Cis- and trans-regulatory divergence assignment

The parental (F0) data sets were first tested for significance of differential gene expression, and offspring (F1) were tested for differential allelic expression at each temperature separately. We applied TMM normalization on read counts of each gene and performed an empirical Bayesian estimation based on negative-binomial GLM to compute gene-wise dispersions [62,63]. The significance of expression divergence was determined by an F-test:

$$Allelic\;Expression\sim \left(\begin{array}{l} allele\;oregonR\\ allele\;Samarkand \end{array}\right)+\varepsilon$$

We further compared the strain-specific allele abundance ratio between F0 and F1 data sets:

$$log_{2}(\frac{O}{O+S})\sim \left(\begin{array}{l} F0\\ F1 \end{array}\right)+\varepsilon$$

Quasi-binominal GLM analysis was performed for each gene and any significant difference between F0 and F1 data set was considered as evidence of *trans* effects (T).

For all statistical analyses applied in F0, F1 and T, *p*-values were adjusted by FDR correction [64] with a nominal cutoff of ≤ 5%. Genes were classified into seven categories by comparing the significance levels from all three tests [24,47]:

1. *Not different*: No significant differential expression in F0 or F1. No significant T.
2. *Cis* only: Significant differential expression in F0 and F1. No significant T.
3. *Trans* only: Significant differential expression in F0, but not in F1. Significant T.
4. *Cis* + *trans*: Significant differential expression in F0 and F1. Significant T. *Cis*- and *trans*-regulatory effects favor expression of the same allele.
5. *Cis* × *trans*: Significant differential expression in F0 and F1. Significant T. *Cis*- and *trans*-regulatory effects favor expression of the different allele.
6. *Compensatory*: Significant differential expression in F1, but not in F0. Significant T. Expression difference caused by *cis*- and *trans*-regulatory components had an opposite direction and perfectly compensate each other such that no expression difference in F0.
7. *Ambiguous*: Significant in only one of differential expression tests in F0, F1 or T. Thus, no explicit *cis*-/*trans*-effect can be detected.

To further confirm our estimates of gene/allelic expression difference, we made a joint estimation using GLM method including allelic difference, temperature, and their interaction:

$$Allelic\;Expression\sim \left(\begin{array}{l} allele\;oregonR\\ allele\;Samarkand \end{array}\right)\times temperature+\varepsilon$$

or

$$log_{2}(\frac{O}{O+S})\sim \left(\begin{array}{l} F0\\ F1 \end{array}\right)\times temperature+\varepsilon$$

### Inbred-hybrid divergence assignment

We evaluated the divergence of total expression (i.e.: ignoring allelic information) between F0 parents and F1 hybrids for each gene and for each temperature, following the previously suggested “mode of inheritance classification” [24,47]. The total gene expression level in F1 flies was estimated as the sum of reads mapped to both parental alleles. TMM normalization followed by a negative-binominal GLM analysis was used to evaluate the expression values of F1 flies between either of the two parents (F0):

$$Total\;Expression\sim \left(\begin{array}{l} F0_{oregonR}\\ F1 \end{array}\right)+\varepsilon$$

or

$$Total\;Expression\sim \left(\begin{array}{l} F0_{samarkand}\\ F1 \end{array}\right)+\varepsilon$$

Genes that have a parent / offspring expression ratio over 1.25-fold and an adjusted *p*-value ≤5% were considered as diverged between F0 and F1 samples and were classified as additive, Oregon R-dominant, Samarkand-dominant, under-dominant, or over-dominant inheritance, based on the magnitude of the difference between total expression in the F1 and in each parental sample (S3 Fig.).

### Enrichment test for transcription-factors and gene sets

Transcription factor (TF) enrichment was tested by comparing the number of target genes for each TF between a test gene-set and all expressed genes. Significance levels were determined by a one tailed hyper-geometric test. We estimated the number of false positives by 1000 random samples, with each sample consisting of the same number of genes as in the test set. The association of multiple transcription factors was investigated by the Limitless-Arity multiple-testing procedure [65]. The significance was calculated using Mann-Whitney U-test and was calibrated by Family-Wise Error Rate. We performed these transcription factor enrichment tests on 149 experimentally verified transcription factors collected from the Drosophila Interactions Database version 2013–07 [66]. The number of regulatory transcription factors in different test sets was used to compare with those in all expressed genes.

Gene-set enrichment analysis was carried out with the software FUNC [67], using all expressed genes as a background gene list. The pathway analysis was performed with the R package “gage” [68]. Genes were mapped to KEGG pathways and pathways enriched with genes of expression divergence were reported.

We have deposited all RNA-Seq raw sequencing reads in NCBI Sequence Read Archive with accession numbers SRP041398 (F0) and SRP041395 (F1). All read-count tables and customized R scripts for statistical analyses have been uploaded to DataDryad.org with accession doi: 10.5061/dryad.pk045.

## Supporting Information

S1 Fig Overview of allele specific expression inference procedure. (TIFF)

S2 Fig The correlation of SNP coverage between two parental data sets. The high correlation suggests that no mapping bias exists.
(TIFF)

S3 Fig Barplots illustrating the six inheritance modes as determined by the comparison of F1 samples to the parental samples. (TIFF)

S4 Fig Test for imprinted genes. The high correlation of the allelic expression profile between F1_A_ and F1_B_ suggests genomic imprinting is absent in D. melanogaster adult female flies. Only for two genes we detected significant imprinting between F1_A_ and F1_B_. ChrU_5299041_5299681.0, which is located on the mtDNA, exhibited a dramatic expression change (indicated by the star symbol), while only minor expression change was detected for CG1275 (indicated by the dot near origin).
(TIFF)

S1 Table Number of read pairs mapped to parental alleles after down-sampling. (DOCX)

S2 Table Summary of genes with expression differences after down-sampling. (DOCX)

S3 Table Summary of expression differences using reads aligned to 500bp at the 3’end of genes after down-sampling. (DOCX)

S4 Table Summary of joint estimated effects of allelic difference, temperature and their interaction on gene expression. (DOCX)

S5 Table Enrichment tests of transcription factors in dominance-swapped genes against all expressed genes. (DOCX)

S6 Table Enrichment tests of combinations of multiple transcription factors in dominance-swapped genes (only top 100 combinations shown here). (DOCX)

S7 Table Top ranked GO terms and core pathway sets from gene set enrichment analyses performed on dominance-swapped genes. (DOCX)

S8 Table Inheritance of gene expression pattern in chorion protein genes. (DOCX)

S9 Table Inheritance mode of gene expression in F1 with two parent-of-origin orders. (DOCX)

S10 Table Inheritance mode of gene expression in F1 with two parent-of-origin orders. (DOCX)

S1 Dataset Detail list of genes in expression differentiation based on joint estimation of allelic difference, temperature and interactions. (XLSX)
