# Correction: Temperature Stress Mediates Decanalization and Dominance of Gene Expression in *Drosophila melanogaster*

**DOI:** 10.1371/journal.pgen.1006079

**Published:** 2016-05-19

**Authors:** The PLOS Genetics Staff

The images for Figs 3 and 4 are incorrectly switched. The image that appears for Fig 3 corresponds to Fig 4 and vice versa. Please view the correct figures and their corresponding legends here. The publisher apologizes for the error.

**Fig 3:**
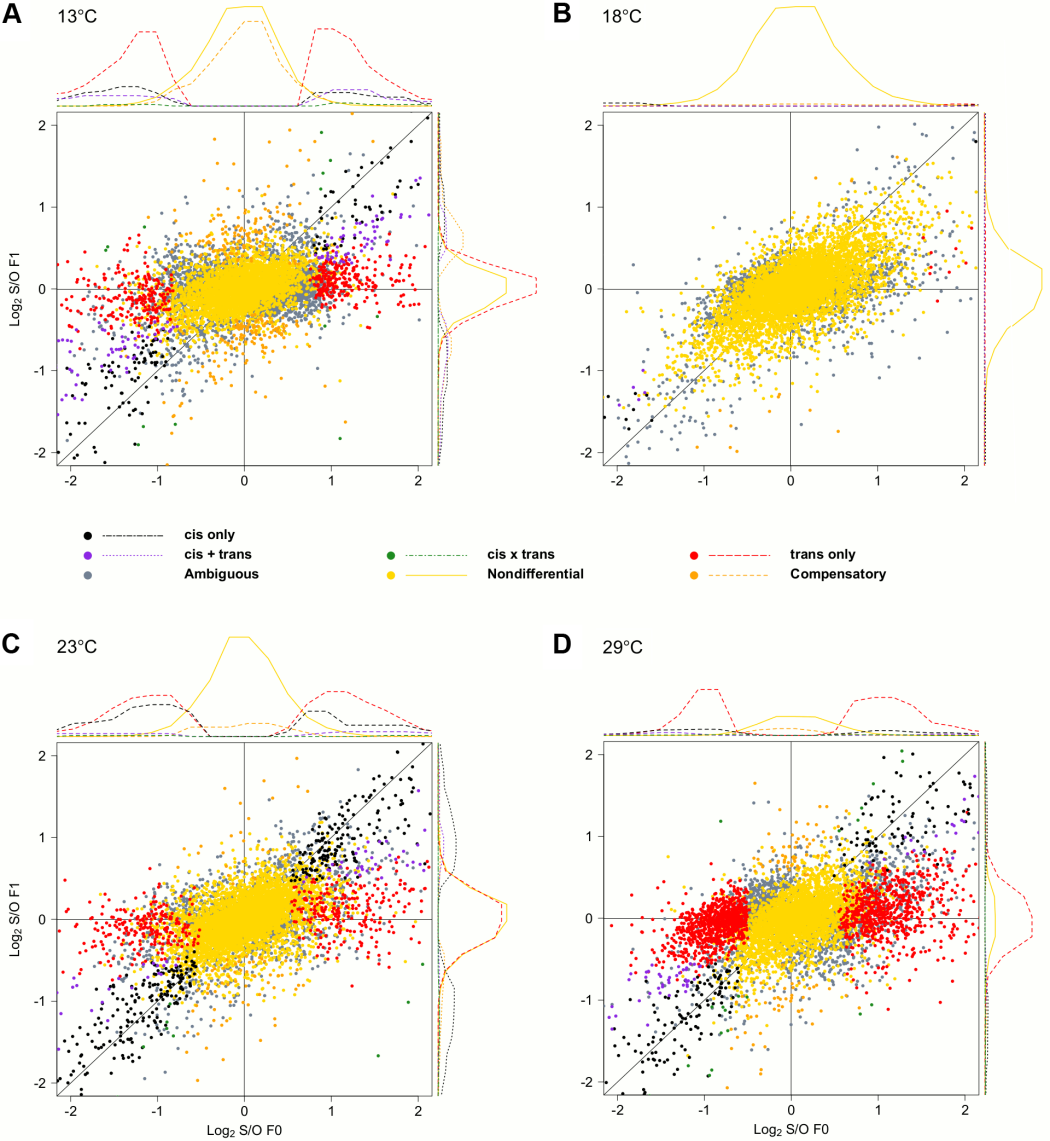
Temperature-dependence of *cis*- and *trans*-effects. Scatter plots contrasting the relative allelic expression levels of parents and F1 offspring. Since the large number of genes makes a quantitative assessment difficult, we also show the density distribution for each class of genes. For representation purposes density distribution of genes with no significant differences in gene expression (yellow) is scaled by 1/10. While at (B) 18°C almost no allelic heterogeneity is present, the number of *cis*- and *trans*-effects increases with more extreme temperatures, (A) 13°C (C) 23°C and (D) 29°C.

**Fig 4:**
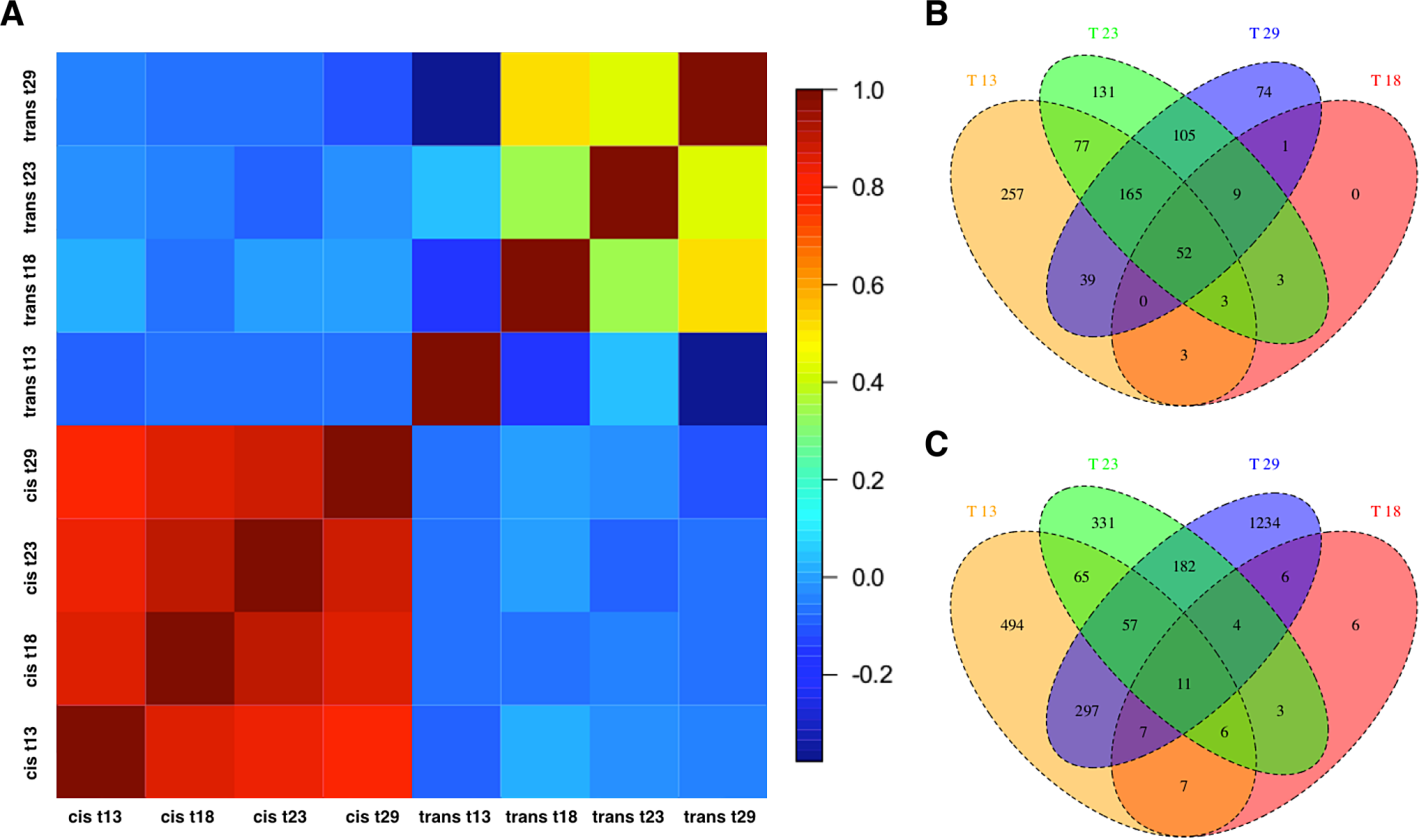
Temperature dependence of *cis*- and *trans*-regulatory differences. (a) Pairwise correlation coefficient matrix (Spearman’s *r*) between *cis*-effects and *trans*-effects across all temperatures. The correlation of *cis*-effects across environments was more similar than the one of trans-effects. (b) Venn Diagram showing the number of *cis*-regulated and (c) *trans*-regulated genes at four different temperatures.
