# Supplementary figures and images for: Temperature Stress Mediates Decanalization and Dominance of Gene Expression in Drosophila melanogaster
Source: PLoS Genet. 2015 Feb 26;11(2):e1004883. doi: 10.1371/journal.pgen.1004883 (PMC4342254; doi:10.1371/journal.pgen.1004883)

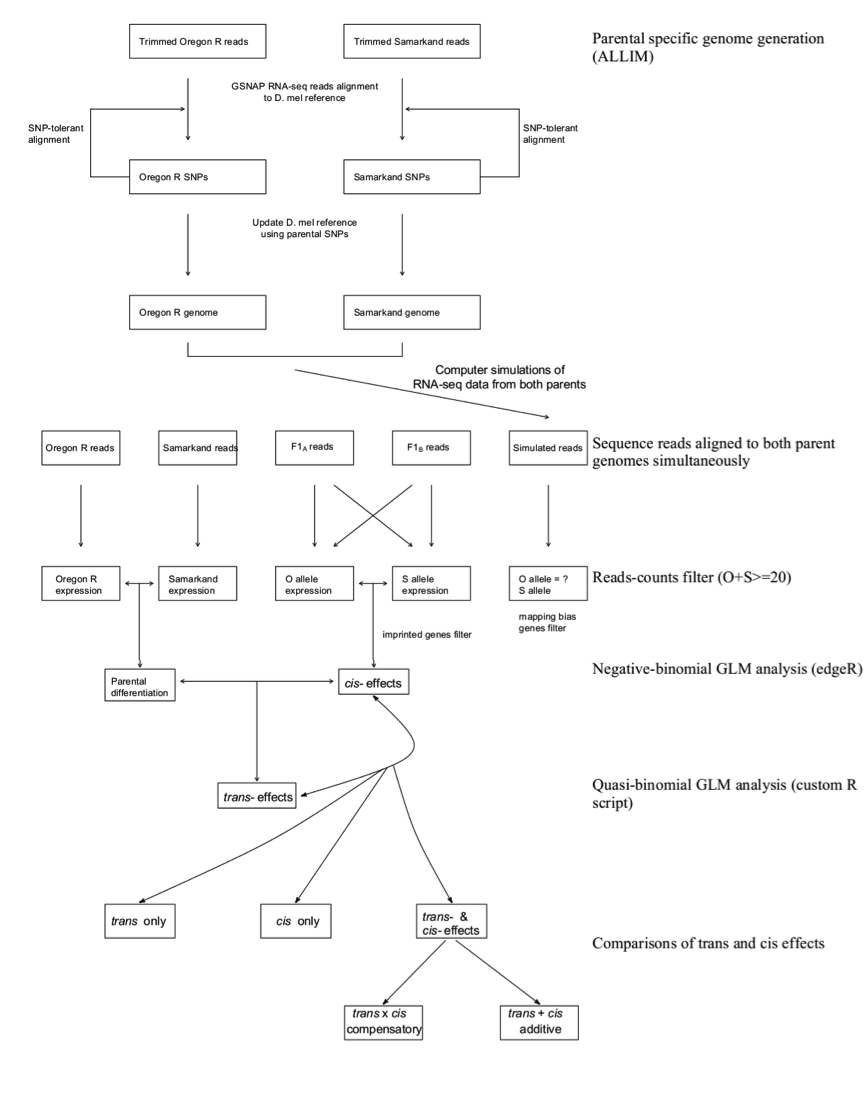

Supplement: S1 Fig — (TIFF) [file pgen.1004883.s001.tiff]

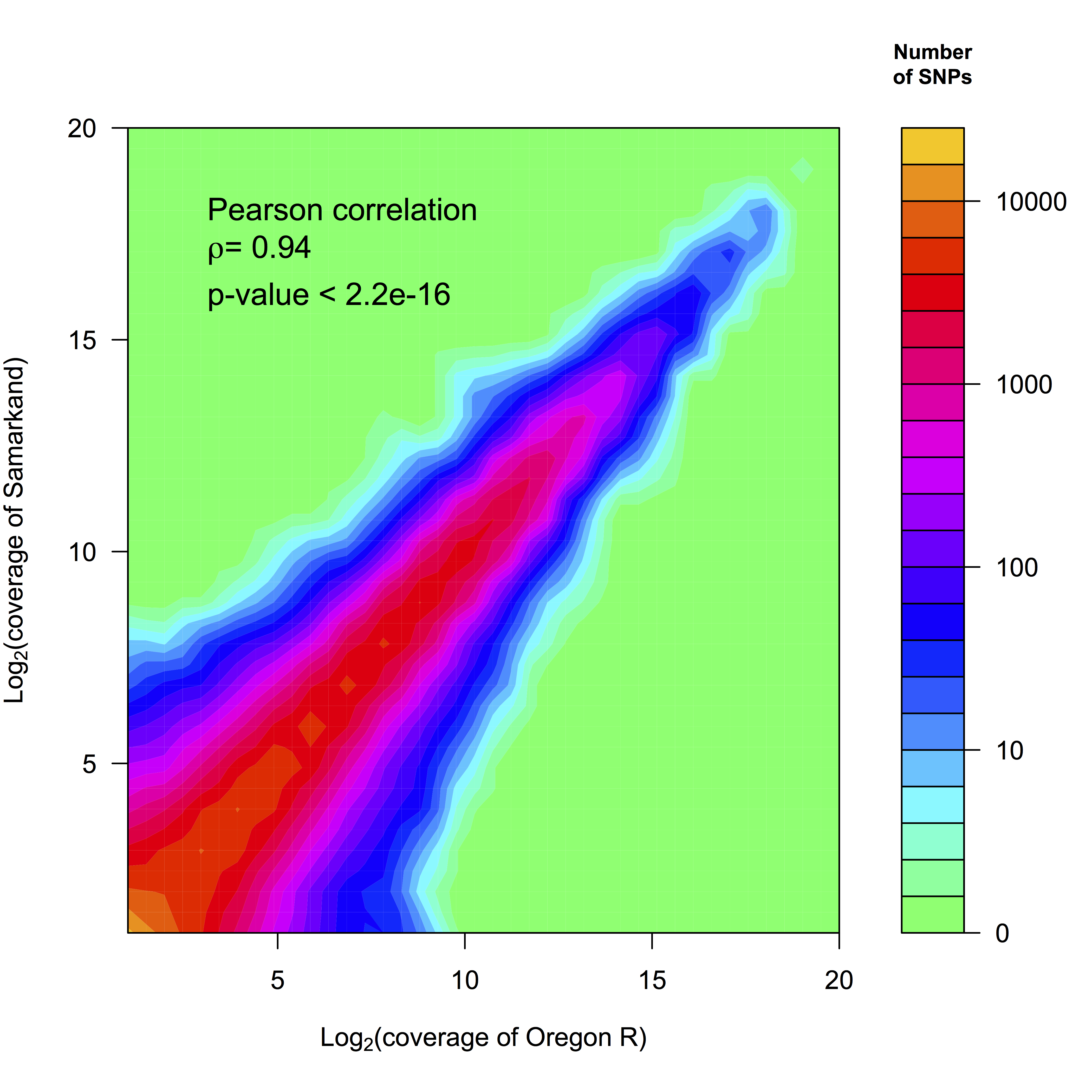

Supplement: S2 Fig — The high correlation suggests that no mapping bias exists. (TIFF) [file pgen.1004883.s002.tiff]

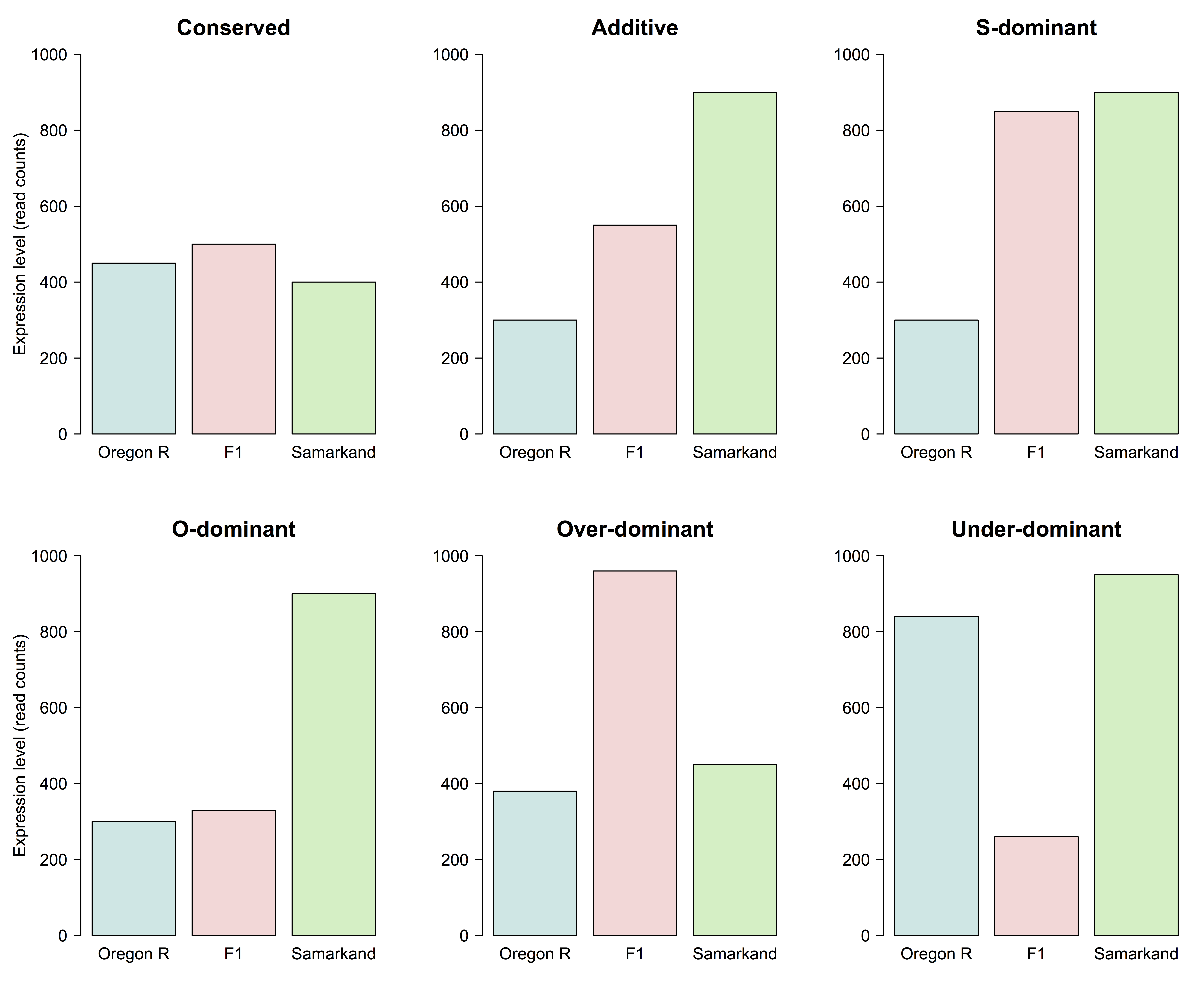

Supplement: S3 Fig — (TIFF) [file pgen.1004883.s003.tiff]

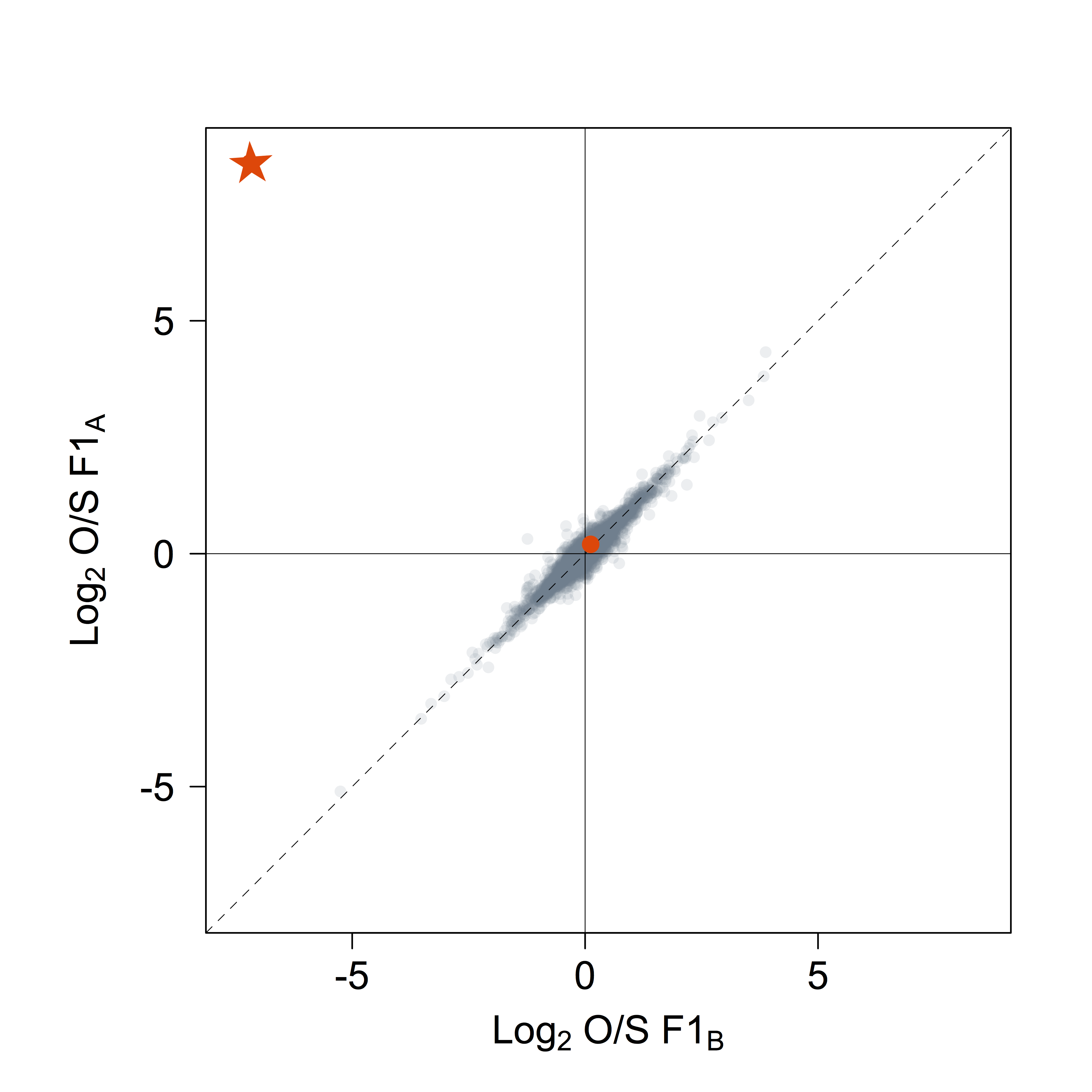

Supplement: S4 Fig — The high correlation of the allelic expression profile between F1A and F1B suggests genomic imprinting is absent in D. melanogaster adult female flies. Only for two genes we detected significant imprinting between F1A and F1B. ChrU_5299041_5299681.0, which is located on the mtDNA, exhibited a dramatic expression change (indicated by the star symbol), while only minor expression change was detected for CG1275 (indicated by the dot near origin). (TIFF) [file pgen.1004883.s004.tiff]
