# Supplementary material for: Temperature Stress Mediates Decanalization and Dominance of Gene Expression in Drosophila melanogaster
Source: PLoS Genet. 2015 Feb 26;11(2):e1004883. doi: 10.1371/journal.pgen.1004883 (PMC4342254; doi:10.1371/journal.pgen.1004883)
Supplement: S1 Table — (DOCX) [file pgen.1004883.s005.docx]

**Table S1 Number of read pairs mapped to parental alleles after down-sampling.**

| **Replicates^a^** | **OregonR** | **Samarkand** | **F1_A_** | | **F1_B_** | |
| --- | --- | --- | --- | --- | --- | --- |
|  | **Allele OregonR** | **Allele Samarkand** | **Allele OregonR** | **Allele Samarkand** | **Allele OregonR** | **Allele Samarkand** |
| t13-rep1 | 9,002,096 | 7,701,519 | 4,801,537 | 4,301,134 | 3,800,619 | 4,100,469 |
| t13-rep2 | 6,800,861 | 7,000,234 | 3,500,141 | 3,300,726 | 3,601,063 | 3,997,083 |
| t13-rep3 | 4,800,623 | 2,500,137 | 3,200,580 | 3,100,309 | 3,400,791 | 3,598,995 |
| t18-rep1 | 6,801,542 | 7,701,498 | 3,500,305 | 3,299,801 | 3,600,947 | 4,100,683 |
| t18-rep2 | 9,000,528 | 2,502,346 | 4,792,525 | 4,300,520 | 3,799,947 | 4,001,664 |
| t18-rep3 | 4,798,960 | 7,002,103 | **3,200,440^b^** | **3,099,920^b^** | 3,401,033 | 3,600,411 |
| t23-rep1 | 4,802,937 | 2,499,463 | 4,801223 | 4,301,365 | 3,601,128 | 4,000,758 |
| t23-rep2 | 9,001,438 | **7,000,557^b^** | 3,200153 | 3,100,317 | 3,799,011 | 4,100,068 |
| t23-rep3 | **6,800,678^c^** | 7,700,181 | 3,500,941 | 3,299,286 | 3,400,853 | 3,600,761 |
| t29-rep1 | 4,796,012 | **6,998,424^c^** | 3,501,491 | 3,299,666 | 3,599,960 | 4,001,835 |
| t29-rep2 | **6,802,281^b^** | 2,500,973 | 3,200,277 | 3,099,667 | 3,399,874 | 3,600,718 |
| t29-rep3 | 9,003,056 | 7,701,151 | 4,801,781 | 4,300,468 | 3,799,606 | 4,099,159 |

a: replicate names correspond to experiment names under the SRA accessions

b: potentially male-contaminated libraries

c: libraries of similar sizes were also removed to balance the analyses in which the male contaminated libraries were removed
