# Supplementary material for: Temperature Stress Mediates Decanalization and Dominance of Gene Expression in Drosophila melanogaster
Source: PLoS Genet. 2015 Feb 26;11(2):e1004883. doi: 10.1371/journal.pgen.1004883 (PMC4342254; doi:10.1371/journal.pgen.1004883)
Supplement: S2 Table — (DOCX) [file pgen.1004883.s006.docx]

**Table S2 Summary of genes with expression differences after down-sampling**

|  |  | **13°C** | **18°C** | **23°C** | **29°C** |
| --- | --- | --- | --- | --- | --- |
| **Divergence in F0** |  | 964 | 84 | 1182 | 2398 |
| **Allelic expression divergence** | Ambiguous (ambig) | 2305 | 1142 | 1046 | 1626 |
|  | Not different (n.s.) | 3633 | 5790 | 5035 | 3549 |
|  | Compensatory | 286 | 17 | 66 | 96 |
|  | *cis* × *trans* | 16 | 0 | 13 | 20 |
|  | *cis* + *trans* | 153 | 12 | 67 | 90 |
|  | *trans* only | 492 | 16 | 437 | 1420 |
|  | *cis* only | 148 | 40 | 366 | 236 |
| **Inheritance modes of gene expression** | Not different (n.s.) | 3397 | 6997 | 6233 | 4860 |
|  | O-dominant  (O-dom) | 140 | 7 | 159 | 2087 |
|  | S-dominant  (S-dom) | 3210 | 13 | 580 | 48 |
|  | Additive | 67 | 0 | 35 | 13 |
|  | Over-  dominant | 81 | 0 | 10 | 6 |
|  | Under-dominant | 122 | 0 | 0 | 3 |
