# Supplementary material for: Temperature Stress Mediates Decanalization and Dominance of Gene Expression in Drosophila melanogaster
Source: PLoS Genet. 2015 Feb 26;11(2):e1004883. doi: 10.1371/journal.pgen.1004883 (PMC4342254; doi:10.1371/journal.pgen.1004883)
Supplement: S3 Table — (DOCX) [file pgen.1004883.s007.docx]

**Table S3 Summary of expression differences using reads aligned to 500bp at the 3’end of genes after down-sampling**

|  |  | **13°C** | **18°C** | **23°C** | **29°C** |
| --- | --- | --- | --- | --- | --- |
| **Divergence in F0** |  | 569 | 61 | 933 | 1117 |
| **Allelic expression divergence** | Ambiguous (ambig) | 817 | 435 | 676 | 749 |
|  | Not different (n.s.) | 2595 | 3422 | 2677 | 2413 |
|  | Compensatory | 82 | 8 | 23 | 53 |
|  | *cis* × *trans* | 10 | 0 | 13 | 9 |
|  | *cis* + *trans* | 50 | 8 | 31 | 42 |
|  | *trans* only | 233 | 5 | 203 | 477 |
|  | *cis* only | 141 | 40 | 295 | 175 |
| **Inheritance modes of gene expression** | Not different (n.s.) | 2361 | 3885 | 3181 | 2967 |
|  | O-dominant  (O-dom) | 95 | 14 | 123 | 874 |
|  | S-dominant  (S-dom) | 1329 | 18 | 546 | 46 |
|  | Additive | 46 | 0 | 56 | 22 |
|  | Over-  dominant | 30 | 0 | 9 | 2 |
|  | Under-dominant | 57 | 1 | 3 | 7 |
