# Supplementary material for: Temperature Stress Mediates Decanalization and Dominance of Gene Expression in Drosophila melanogaster
Source: PLoS Genet. 2015 Feb 26;11(2):e1004883. doi: 10.1371/journal.pgen.1004883 (PMC4342254; doi:10.1371/journal.pgen.1004883)
Supplement: S4 Table — (DOCX) [file pgen.1004883.s008.docx]

**Table S4 Summary of expression differences after removing samples that potentially included a small number of males**

|  |  | **13°C** | **18°C** | **23°C** | **29°C** |
| --- | --- | --- | --- | --- | --- |
| **Divergence in F0** |  | 572 | 61 | 738 | 499 |
| **Allelic expression divergence** | Ambiguous (ambig) | 824 | 289 | 648 | 577 |
|  | Not different (n.s.) | 2587 | 3574 | 2820 | 2990 |
|  | Compensatory | 82 | 4 | 19 | 28 |
|  | *cis* × *trans* | 10 | 0 | 8 | 1 |
|  | *cis* + *trans* | 50 | 6 | 23 | 15 |
|  | *trans* only | 224 | 4 | 152 | 161 |
|  | *cis* only | 142 | 42 | 249 | 147 |
| **Inheritance modes of gene expression** | Not different (n.s.) | 2361 | 3886 | 3540 | 3342 |
|  | O-dominant  (O-dom) | 95 | 14 | 124 | 554 |
|  | S-dominant  (S-dom) | 1330 | 19 | 219 | 16 |
|  | Additive | 46 | 0 | 31 | 5 |
|  | Over-  dominant | 30 | 0 | 3 | 1 |
|  | Under-dominant | 57 | 0 | 2 | 1 |

See Table S1 for the excluded libraries; the analysis is based only on those reads that mapped to the 500bp at the 3’ end of the expressed genes.
