# Supplementary material for: Temperature Stress Mediates Decanalization and Dominance of Gene Expression in Drosophila melanogaster
Source: PLoS Genet. 2015 Feb 26;11(2):e1004883. doi: 10.1371/journal.pgen.1004883 (PMC4342254; doi:10.1371/journal.pgen.1004883)
Supplement: S5 Table — (DOCX) [file pgen.1004883.s009.docx]

**Table S5 Summary of joint estimated effects of allelic difference, temperature and their interaction on gene expression**

|  | Allelic/Gene differentiation^a^ | Temperature^a^ | Interaction^a^ |
| --- | --- | --- | --- |
| F0 | 1,229 | 5,308 | 1,454 |
| F1 (*cis*-effects) | 480 | 5,238 | 22 |
| *Trans*-effects | 4,994 | 4,778 | 4,334 |

a: A detail list of genes with coefficients and p-values can be found in Dataset S1
