# Supplementary material for: Temperature Stress Mediates Decanalization and Dominance of Gene Expression in Drosophila melanogaster
Source: PLoS Genet. 2015 Feb 26;11(2):e1004883. doi: 10.1371/journal.pgen.1004883 (PMC4342254; doi:10.1371/journal.pgen.1004883)
Supplement: S6 Table — (DOCX) [file pgen.1004883.s010.docx]

**Table S6 Enrichment tests of transcription factors in dominance-swapped genes against all expressed genes**

| **Transcription factors** | **# of targets in swapped genes** | **# of targets in all expressed genes** | ***p*-value** | **FDR** |
| --- | --- | --- | --- | --- |
| *Cp190* | 1044 | 5063 | 2.46E-06 | 0 |
| *sens* | 908 | 4303 | 5.88E-07 | 0 |
| *Myb* | 883 | 4162 | 3.70E-07 | 0 |
| *trx* | 1073 | 4937 | 4.44E-16 | 0 |
| *Med* | 775 | 3645 | 6.53E-06 | 0 |
| *BEAF-32* | 1053 | 4914 | 1.46E-12 | 0 |
| *phol* | 622 | 2858 | 7.20E-06 | 0 |
| *Chro* | 1124 | 5242 | 1.11E-15 | 0 |
| *dl* | 1054 | 4991 | 4.62E-10 | 0 |
| *D* | 827 | 3947 | 2.94E-05 | 9.09E-05 |
| *mip120* | 788 | 3726 | 1.29E-05 | 1.00E-04 |
| *CtBP* | 202 | 900 | 5.92E-03 | 3.22E-02 |
| *ftz* | 24 | 75 | 5.70E-03 | 3.28E-02 |
