# Supplementary material for: Temperature Stress Mediates Decanalization and Dominance of Gene Expression in Drosophila melanogaster
Source: PLoS Genet. 2015 Feb 26;11(2):e1004883. doi: 10.1371/journal.pgen.1004883 (PMC4342254; doi:10.1371/journal.pgen.1004883)
Supplement: S7 Table — (DOCX) [file pgen.1004883.s011.docx]

**Table S7 Enrichment tests of combinations of multiple transcription factors in dominance-swapped genes (only top 100 combinations shown here)**

| **Rank** | **Adjusted *p*-value** | **Combination** | **Arity** | **# of target rows** | **z-score** |
| --- | --- | --- | --- | --- | --- |
| 1 | 2.98E-50 | *BEAF-32,Chro* | 2 | 934 | 15.756 |
| 2 | 3.76E-47 | *BEAF-32,Chro,Cp190* | 3 | 875 | 15.298 |
| 3 | 2.09E-46 | *Chro,Cp190* | 2 | 916 | 15.185 |
| 4 | 2.82E-45 | *BEAF-32,Chro,trx* | 3 | 884 | 15.014 |
| 5 | 4.12E-45 | *BEAF-32,Cp190* | 2 | 880 | 14.989 |
| 6 | 1.76E-43 | *Chro,Cp190,trx* | 3 | 877 | 14.737 |
| 7 | 4.04E-43 | *BEAF-32,Chro,Cp190,trx* | 4 | 843 | 14.681 |
| 8 | 4.75E-42 | *BEAF-32* | 1 | 956 | 14.513 |
| 9 | 7.07E-42 | *BEAF-32,Cp190,trx* | 3 | 846 | 14.486 |
| 10 | 1.16E-41 | *Chro* | 1 | 1019 | 14.452 |
| 11 | 2.08E-40 | *BEAF-32,trx* | 2 | 896 | 14.252 |
| 12 | 3.71E-40 | *Chro,trx* | 2 | 939 | 14.211 |
| 13 | 6.16E-40 | *Cp190,trx* | 2 | 888 | 14.175 |
| 14 | 7.67E-40 | *BEAF-32,Chro,dl* | 3 | 834 | 14.16 |
| 15 | 3.70E-39 | *Chro,Cp190,sens* | 3 | 713 | 14.049 |
| 16 | 3.81E-38 | *BEAF-32,Chro,Cp190,dl* | 4 | 800 | 13.883 |
| 17 | 4.24E-38 | *BEAF-32,Chro,sens* | 3 | 722 | 13.875 |
| 18 | 3.00E-37 | *BEAF-32,Chro,Cp190,sens* | 4 | 690 | 13.734 |
| 19 | 8.53E-37 | *BEAF-32,Cp190,sens* | 3 | 691 | 13.658 |
| 20 | 1.39E-36 | *Chro,sens* | 2 | 761 | 13.623 |
| 21 | 1.75E-36 | *BEAF-32,Cp190,dl* | 3 | 804 | 13.606 |
| 22 | 2.30E-36 | *Chro,Cp190,dl* | 3 | 825 | 13.586 |
| 23 | 6.52E-36 | *BEAF-32,Chro,dl,trx* | 4 | 805 | 13.509 |
| 24 | 7.04E-36 | *BEAF-32,Chro,Cp190,dl,trx* | 5 | 780 | 13.504 |
| 25 | 1.87E-35 | *Cp190,sens* | 2 | 727 | 13.432 |
| 26 | 2.66E-35 | *Chro,Cp190,dl,trx* | 4 | 802 | 13.406 |
| 27 | 3.16E-35 | *Cp190* | 1 | 949 | 13.393 |
| 28 | 5.74E-35 | *Chro,Cp190,sens,trx* | 4 | 687 | 13.348 |
| 29 | 6.96E-35 | *BEAF-32,sens* | 2 | 732 | 13.334 |
| 30 | 1.03E-34 | *BEAF-32,Cp190,dl,trx* | 4 | 783 | 13.305 |
| 31 | 6.94E-34 | *Chro,dl* | 2 | 882 | 13.161 |
| 32 | 1.01E-33 | *BEAF-32,Chro,sens,trx* | 4 | 690 | 13.133 |
| 33 | 1.80E-33 | *BEAF-32,Chro,dl,sens* | 4 | 661 | 13.089 |
| 34 | 1.94E-33 | *BEAF-32,dl* | 2 | 851 | 13.084 |
| 35 | 1.96E-33 | *Cp190,sens,trx* | 3 | 693 | 13.083 |
| 36 | 4.21E-33 | *Chro,sens,trx* | 3 | 718 | 13.024 |
| 37 | 8.97E-33 | *BEAF-32,Chro,Cp190,sens,trx* | 5 | 668 | 12.967 |
| 38 | 2.21E-32 | *Cp190,dl,trx* | 3 | 810 | 12.897 |
| 39 | 2.42E-32 | *BEAF-32,Cp190,sens,trx* | 4 | 669 | 12.89 |
| 40 | 2.87E-32 | *Chro,Cp190,dl,sens* | 4 | 659 | 12.877 |
| 41 | 4.44E-32 | *BEAF-32,Chro,Cp190,dl,sens* | 5 | 642 | 12.843 |
| 42 | 5.02E-32 | *BEAF-32,dl,trx* | 3 | 815 | 12.834 |
| 43 | 8.23E-32 | *BEAF-32,sens,trx* | 3 | 695 | 12.796 |
| 44 | 1.20E-31 | *BEAF-32,Cp190,dl,sens* | 4 | 643 | 12.766 |
| 45 | 5.33E-31 | *BEAF-32,Chro,Cp190,Myb* | 4 | 726 | 12.65 |
| 46 | 9.23E-31 | *Chro,dl,trx* | 3 | 843 | 12.606 |
| 47 | 9.80E-31 | *Chro,dl,sens* | 3 | 689 | 12.602 |
| 48 | 1.43E-30 | *Cp190,dl* | 2 | 840 | 12.572 |
| 49 | 1.73E-30 | *BEAF-32,Cp190,Myb* | 3 | 728 | 12.557 |
| 50 | 2.87E-30 | *BEAF-32,dl,sens* | 3 | 670 | 12.517 |
| 51 | 4.10E-30 | *BEAF-32,Chro,Myb,trx* | 4 | 725 | 12.488 |
| 52 | 4.80E-30 | *trx* | 1 | 973 | 12.476 |
| 53 | 5.14E-30 | *Chro,Cp190,dl,sens,trx* | 5 | 642 | 12.47 |
| 54 | 6.40E-30 | *BEAF-32,Chro,Myb* | 3 | 749 | 12.453 |
| 55 | 9.31E-30 | *BEAF-32,Chro,Cp190,Myb,trx* | 5 | 711 | 12.423 |
| 56 | 1.63E-29 | *BEAF-32,Myb,trx* | 3 | 727 | 12.378 |
| 57 | 1.80E-29 | *BEAF-32,Cp190,Myb,trx* | 4 | 712 | 12.37 |
| 58 | 2.06E-29 | *Chro,Cp190,Myb* | 3 | 757 | 12.359 |
| 59 | 2.43E-29 | *BEAF-32,Chro,mip120* | 3 | 691 | 12.346 |
| 60 | 4.12E-29 | *BEAF-32,Myb* | 2 | 752 | 12.303 |
| 61 | 4.60E-29 | *Chro,Cp190,Myb,trx* | 4 | 736 | 12.294 |
| 62 | 4.74E-29 | *BEAF-32,Chro,dl,sens,trx* | 5 | 642 | 12.292 |
| 63 | 5.21E-29 | *BEAF-32,Chro,Cp190,dl,sens,trx* | 6 | 627 | 12.284 |
| 64 | 7.52E-29 | *Cp190,dl,sens* | 3 | 668 | 12.255 |
| 65 | 1.03E-28 | *Cp190,Myb,trx* | 3 | 739 | 12.229 |
| 66 | 1.33E-28 | *Cp190,Myb* | 2 | 762 | 12.208 |
| 67 | 1.36E-28 | *BEAF-32,Cp190,dl,sens,trx* | 5 | 628 | 12.207 |
| 68 | 1.62E-28 | *BEAF-32,mip120* | 2 | 694 | 12.192 |
| 69 | 2.37E-28 | *sens,trx* | 2 | 735 | 12.161 |
| 70 | 2.70E-28 | *Cp190,dl,sens,trx* | 4 | 647 | 12.151 |
| 71 | 1.45E-27 | *BEAF-32,Chro,mip120,trx* | 4 | 666 | 12.013 |
| 72 | 1.77E-27 | *BEAF-32,Chro,Cp190,mip120* | 4 | 679 | 11.996 |
| 73 | 2.81E-27 | *Chro,dl,sens,trx* | 4 | 665 | 11.958 |
| 74 | 2.94E-27 | *Chro,Myb,trx* | 3 | 759 | 11.954 |
| 75 | 3.15E-27 | *BEAF-32,dl,sens,trx* | 4 | 647 | 11.948 |
| 76 | 5.58E-27 | *BEAF-32,Cp190,mip120* | 3 | 681 | 11.9 |
| 77 | 5.67E-27 | *BEAF-32,mip120,trx* | 3 | 668 | 11.899 |
| 78 | 7.88E-27 | *Chro,mip120* | 2 | 718 | 11.872 |
| 79 | 1.18E-26 | *Chro,Myb* | 2 | 793 | 11.838 |
| 80 | 1.25E-26 | *Myb,trx* | 2 | 763 | 11.833 |
| 81 | 2.90E-26 | *Chro,Cp190,Myb,sens* | 4 | 607 | 11.762 |
| 82 | 3.66E-26 | *Cp190,Myb,sens* | 3 | 609 | 11.742 |
| 83 | 4.48E-26 | *BEAF-32,Chro,Cp190,Myb,dl* | 5 | 676 | 11.725 |
| 84 | 5.00E-26 | *BEAF-32,Chro,Cp190,mip120,trx* | 5 | 658 | 11.716 |
| 85 | 5.73E-26 | *Myb* | 1 | 803 | 11.705 |
| 86 | 8.44E-26 | *BEAF-32,Cp190,Myb,dl* | 4 | 677 | 11.672 |
| 87 | 9.47E-26 | *BEAF-32,Cp190,mip120,trx* | 4 | 659 | 11.662 |
| 88 | 1.04E-25 | *Chro,mip120,trx* | 3 | 685 | 11.654 |
| 89 | 1.19E-25 | *BEAF-32,Chro,Cp190,Myb,sens* | 5 | 588 | 11.643 |
| 90 | 1.30E-25 | *BEAF-32,Chro,Myb,dl* | 4 | 688 | 11.635 |
| 91 | 1.98E-25 | *mip120* | 1 | 723 | 11.599 |
| 92 | 3.98E-25 | *BEAF-32,Chro,Cp190,Myb,dl,trx* | 6 | 666 | 11.539 |
| 93 | 4.55E-25 | *BEAF-32,Chro,Myb,sens* | 4 | 600 | 11.527 |
| 94 | 4.80E-25 | *BEAF-32,Myb,dl* | 3 | 690 | 11.523 |
| 95 | 5.59E-25 | *Chro,Cp190,mip120* | 3 | 701 | 11.51 |
| 96 | 6.77E-25 | *BEAF-32,Chro,Myb,dl,trx* | 5 | 674 | 11.493 |
| 97 | 7.18E-25 | *BEAF-32,Chro,Myb,mip120* | 4 | 648 | 11.488 |
| 98 | 7.32E-25 | *Chro,Myb,sens* | 3 | 624 | 11.486 |
| 99 | 7.41E-25 | *Myb,sens* | 2 | 628 | 11.485 |
| 100 | 7.45E-25 | *BEAF-32,Cp190,Myb,dl,trx* | 5 | 667 | 11.485 |
