# Supplementary material for: Temperature Stress Mediates Decanalization and Dominance of Gene Expression in Drosophila melanogaster
Source: PLoS Genet. 2015 Feb 26;11(2):e1004883. doi: 10.1371/journal.pgen.1004883 (PMC4342254; doi:10.1371/journal.pgen.1004883)
Supplement: S8 Table — (DOCX) [file pgen.1004883.s012.docx]

**Table S8 Top ranked GO terms and core pathway sets from gene set enrichment analyses performed on dominance-swapped genes**

| **GO / KEGG** | **GO / Pathway description** | **Adjusted *p*-value** |
| --- | --- | --- |
| GO:0006412 | translation | 1.69E-06 |
| GO:0048790 | maintenance of presynaptic active zone structure | 0.00142 |
| GO:0048856 | anatomical structure development | 0.00146 |
| GO:0044767 | single-organism developmental process | 0.00426 |
| GO:0006626 | protein targeting to mitochondrion | 0.00587 |
| GO:0007589 | body fluid secretion | 0.00732 |
| GO:0035149 | lumen formation, open tracheal system | 0.00911 |
| GO:0007052 | mitotic spindle organization | 0.01425 |
| GO:0015698 | inorganic anion transport | 0.01660 |
| GO:0016485 | protein processing | 0.01660 |
| GO:0030011 | maintenance of cell polarity | 0.01679 |
| GO:0050821 | protein stabilization | 0.01679 |
| GO:0046488 | phosphatidylinositol metabolic process | 0.01870 |
| GO:0000398 | mRNA splicing, via spliceosome | 0.02083 |
| GO:0015991 | ATP hydrolysis coupled proton transport | 0.02279 |
| GO:0022613 | ribonucleoprotein complex biogenesis | 0.02311 |
| GO:0006457 | protein folding | 0.02336 |
| GO:0048731 | system development | 0.02501 |
| GO:0006465 | signal peptide processing | 0.02504 |
| GO:0006729 | tetrahydrobiopterin biosynthetic process | 0.02504 |
| GO:0019896 | axon transport of mitochondrion | 0.02504 |
| GO:0042180 | cellular ketone metabolic process | 0.02716 |
| GO:0006614 | SRP-dependent cotranslational protein targeting to membrane | 0.02908 |
| GO:0006754 | ATP biosynthetic process | 0.02908 |
| GO:0009612 | response to mechanical stimulus | 0.02908 |
| GO:0006458 | 'de novo' protein folding | 0.03012 |
| GO:0043524 | negative regulation of neuron apoptotic process | 0.03012 |
| GO:0031122 | cytoplasmic microtubule organization | 0.03240 |
| GO:0006631 | fatty acid metabolic process | 0.03428 |
| GO:0007275 | multicellular organismal development | 0.03711 |
| GO:0003331 | positive regulation of extracellular matrix constituent secretion | 0.03775 |
| GO:0006433 | prolyl-tRNA aminoacylation | 0.03775 |
| GO:0006612 | protein targeting to membrane | 0.03775 |
| GO:0006857 | oligopeptide transport | 0.03775 |
| GO:0007021 | tubulin complex assembly | 0.03775 |
| GO:0007396 | suture of dorsal opening | 0.03775 |
| GO:0009052 | pentose-phosphate shunt, non-oxidative branch | 0.03775 |
| GO:0009221 | pyrimidine deoxyribonucleotide biosynthetic process | 0.03775 |
| GO:0015701 | bicarbonate transport | 0.03775 |
| GO:0016559 | peroxisome fission | 0.03775 |
| GO:0033522 | histone H2A ubiquitination | 0.03775 |
| GO:0034394 | protein localization to cell surface | 0.03775 |
| GO:2000035 | regulation of stem cell division | 0.03775 |
| GO:0007338 | single fertilization | 0.04496 |
| GO:0072348 | sulfur compound transport | 0.04496 |
| GO:0006726 | eye pigment biosynthetic process | 0.04805 |
| GO:0008033 | tRNA processing | 0.04805 |
| dme03010 | Ribosome | 5.00E-35 |
| dme03040 | Spliceosome | 6.48E-18 |
| dme04141 | Protein processing in endoplasmic reticulum | 2.26E-09 |
| dme04120 | Ubiquitin mediated proteolysis | 2.41E-08 |
| dme03013 | RNA transport | 5.86E-08 |
| dme00240 | Pyrimidine metabolism | 7.59E-07 |
| dme03015 | mRNA surveillance pathway | 1.16E-05 |
| dme03018 | RNA degradation | 0.0034 |
