# Supplementary material for: Temperature Stress Mediates Decanalization and Dominance of Gene Expression in Drosophila melanogaster
Source: PLoS Genet. 2015 Feb 26;11(2):e1004883. doi: 10.1371/journal.pgen.1004883 (PMC4342254; doi:10.1371/journal.pgen.1004883)
Supplement: S9 Table — (DOCX) [file pgen.1004883.s013.docx]

**Table S9 Inheritance of gene expression pattern in chorion protein genes**

| **Name** | **Symbol** | **FlyBase ID** | **Inheritance of gene expression** | | | |
| --- | --- | --- | --- | --- | --- | --- |
|  |  |  | **13°C** | **18°C** | **23°C** | **29°C** |
| Chorion protein 15 | *Cp15* | FBgn0000355 | n.s.^a^ | n.s. | n.s. | n.s. |
| Chorion protein 16 | *Cp16* | FBgn0000356 | n.s. | n.s. | n.s. | n.s. |
| Chorion protein 18 | *Cp18* | FBgn0000357 | n.s. | n.s. | n.s. | n.s. |
| Chorion protein 19 | *Cp19* | FBgn0000358 | n.s. | n.s. | O-dom^b^ | n.s. |
| Chorion protein 36 | *Cp36* | FBgn0000359 | n.s. | n.s. | n.s. | n.s. |
| Chorion protein 38 ^c^ | *Cp38* | FBgn0000360 | / | / | / | / |
| Chorion protein 70 ^c^ | *Cp70* | FBgn0000361 | / | / | / | / |
| Chorion factor 2 | *Cf20* | FBgn0000286 | n.s. | n.s. | n.s. | n.s. |
| Chorion protein a at 7F | *Cp7Fa* | FBgn0014464 | n.s. | n.s. | n.s. | O-dom |
| Chorion protein b at 7F | *Cp7Fb* | FBgn0014465 | n.s. | n.s. | n.s. | O-dom |
| Chorion protein c at 7F | *Cp7Fc* | FBgn0014466 | n.s. | n.s. | n.s. | n.s. |

a.: not different

b: Oregon R-dominant

c: does not have allelic difference
