# Supplementary material for: Temperature Stress Mediates Decanalization and Dominance of Gene Expression in Drosophila melanogaster
Source: PLoS Genet. 2015 Feb 26;11(2):e1004883. doi: 10.1371/journal.pgen.1004883 (PMC4342254; doi:10.1371/journal.pgen.1004883)
Supplement: S10 Table — (DOCX) [file pgen.1004883.s014.docx]

**Table S10 Inheritance mode of gene expression in F1 with two parent-of-origin orders**

|  | **13°C** | | **18°C** | | **23°C** | | **29°C** | |
| --- | --- | --- | --- | --- | --- | --- | --- | --- |
|  | F1_A_ | F1_B_ | F1_A_ | F1_B_ | F1_A_ | F1_B_ | F1_A_ | F1_B_ |
| Not different (n.s.) | 5215 | 4267 | 7177 | 7187 | 6992 | 6477 | 4758 | 6082 |
| O-dominant  (O-dom) | 37 | 70 | 8 | 0 | 50 | 199 | 2368 | 1081 |
| S-dominant  (S-dom) | 1906 | 2735 | 2 | 2 | 145 | 474 | 45 | 20 |
| Additive | 22 | 29 | 0 | 0 | 2 | 30 | 8 | 6 |
| Over-  dominant | 3 | 33 | 0 | 0 | 0 | 8 | 4 | 0 |
| Under-dominant | 6 | 55 | 2 | 0 | 0 | 1 | 6 | 0 |
