# Supplementary material for: Correction: Temperature Stress Mediates Decanalization and Dominance of Gene Expression in Drosophila melanogaster
Source: PLoS Genet. 2015 Jun 15;11(6):e1005315. doi: 10.1371/journal.pgen.1005315 (PMC4468180; doi:10.1371/journal.pgen.1005315)
Supplement: S2 File — (PDF) [file pgen.1005315.s002.pdf]

RESEARCH ARTICLE

Temperature Stress Mediates Decanalization and Dominance of Gene Expression in *Drosophila melanogaster*

Jun Chen, Viola Nolte, Christian Schlötterer*

Institut für Populationsgenetik, Vienna, Austria

* christian.schloetterer@vetmeduni.ac.at

OPEN ACCESS

Citation: Chen J, Nolte V, Schlötterer C (2015) Temperature Stress Mediates Decanalization and Dominance of Gene Expression in *Drosophila melanogaster*. PLoS Genet 11(2): e1004883. doi:10.1371/journal.pgen.1004883

Editor: Greg Gibson, Georgia Institute of Technology, UNITED STATES

Received: July 10, 2014

Accepted: November 10, 2014

Published: February 26, 2015

Copyright: © 2015 Chen et al. This is an open access article distributed under the terms of the Creative Commons Attribution License, which permits unrestricted use, distribution, and reproduction in any medium, provided the original author and source are credited.

Data Availability Statement: We have deposited all RNA-Seq raw sequencing reads in NCBI Sequence Read Archive with accession numbers SRP041398 (F0) and SRP041395 (F1). All read-count tables and customized R scripts for statistical analyses have been uploaded to DataDryad.org with accession doi: 10.5061/dryad.pk045

Funding: This work has been supported by the Austrian Science Funds (FWF P22834). The funders had no role in study design, data collection and analysis, decision to publish, or preparation of the manuscript.

Abstract

The regulatory architecture of gene expression remains an area of active research. Here, we studied how the interplay of genetic and environmental variation affects gene expression by exposing *Drosophila melanogaster* strains to four different developmental temperatures. At 18°C we observed almost complete canalization with only very few allelic effects on gene expression. In contrast, at the two temperature extremes, 13°C and 29°C a large number of allelic differences in gene expression were detected due to both *cis*- and *trans*-regulatory effects. Allelic differences in gene expression were mainly dominant, but for up to 62% of the genes the dominance swapped between 13 and 29°C. Our results are consistent with stabilizing selection causing buffering of allelic expression variation in non-stressful environments. We propose that decanalization of gene expression in stressful environments is not only central to adaptation, but may also contribute to genetic disorders in human populations.

Author Summary

Gene expression is the most direct link between the genetically encoded information and the phenotype. We analyzed the patterns of gene expression in two different *D. melanogaster* genotypes and their offspring at four different temperatures to determine if gene expression regulation is modulated by temperature. Interestingly, we find that at the intermediate temperature (18°C) alleles from both genotypes have very similar gene expression, suggesting a strong canalization of gene expression despite substantial genetic differences. More extreme temperatures break this canalization and result in many differently expressed genes, caused mainly by *trans*-acting factors. Most of the expression differences are non-additive, with a swap in dominance between the two extreme temperatures.

Introduction

Canalization, the buffering of a phenotype against environmental or genetic perturbation, has been independently suggested by Schmalhausen [1] and Waddington [2]. Most models of

Competing Interests: The authors have declared that no competing interests exist.

canalization assume that canalization evolves in a response to stabilizing selection (reviewed in [3,4]), but using simulations Siegal and Bergman (2002) found that canalization can also be achieved by regulatory networks even in absence of stabilizing selection [3]. Conversely, genetic or environmental perturbations can result in the loss of canalization ("decanalization") and thus the release of previously hidden or "cryptic" genetic variation. The perhaps best example for such decanalization induced by strong genetic perturbations comes from a series of experiments focusing on impaired function of the chaperone HSP90 [5–7], but other examples have been described in Drosophila [8–15].

Phenotypes are determined by spatial and temporal patterns of gene expression and environmental and genetic variation has been documented to affect gene expression. How the interplay of genetic and environmental variation affects gene expression can be studied with sequencing based expression profiling (RNA-Seq). Many studies have used expression profiling to study genetically diverged individuals/populations at two environments (e.g.:[16–20]), but the canalization of gene expression across the entire transcriptome still remains understudied. Allele specific gene expression analysis in two inbred lines and their progeny is a popular approach to gain insight into the regulatory architecture of gene expression [21–24]. Here, we expose two *D. melanogaster* strains to four different temperatures to determine how temperature stress affects gene expression. Specifically, we analyze the canalization of gene expression of two genotypes. Furthermore, we expand the analysis of canalization by dissecting the *cis*-and *trans*-effects at different temperatures.

Results

The two inbred *D. melanogaster* strains Oregon R (O) and Samarkand (S) were crossed and eggs laid at 23°C were transferred to four different development temperatures (13°C, 18°C, 23°C and 29°C, Fig. 1). We measured gene expression by performing RNA-Seq on three replicates of each of the four genotypes in adult females. Paired-end reads were mapped to reference genomes that were modified to avoid preferential mapping of reads from one of the two parental genomes (see also Material and Methods). Since we were interested in allelic differences in gene expression, only those reads were further processed, which mapped unambiguously to one of the two modified reference genomes (see Table 1 and Material and Methods).

Almost complete absence of imprinting in Drosophila

Comparing the F1 individuals from crosses of Samarkand with Oregon R in both directions we tested for the presence of imprinting in our experiments. Consistent with previous results [25–27], levels of genes expression were very similar between the two genotypes, indicating the absence of imprinting in *D. melanogaster*. Only two genes, chrU_5299041_5299681.0 and CG1275, showed significant parent-of-origin effect (FDR = 5.6e-19 and 0.02, S4 Fig.). While the expression difference of CG1275 was small, chrU_5299041_5299681.0 encoding the cytochrome b gene differed more than 5.2e4-fold between $F1_A$ and $F1_B$. This result indicates a different mitochondrial gene expression rather than imprinting. After excluding both genes, we combined the data from F1 offspring from crosses in both directions.

Decanalization of gene expression between parents

7,189 genes were expressed in parents and offspring and showed allelic differences between the parental strains Oregon R and Samarkand. Comparing the gene expression profiles between the two parental strains we observed a striking difference in gene expression divergence for flies grown at different temperatures (13°C, 18°C, 23°C and 29°C) (Table 2). At 18°C only 92 genes (1.2%) differed significantly in gene expression between Oregon R and Samarkand. In

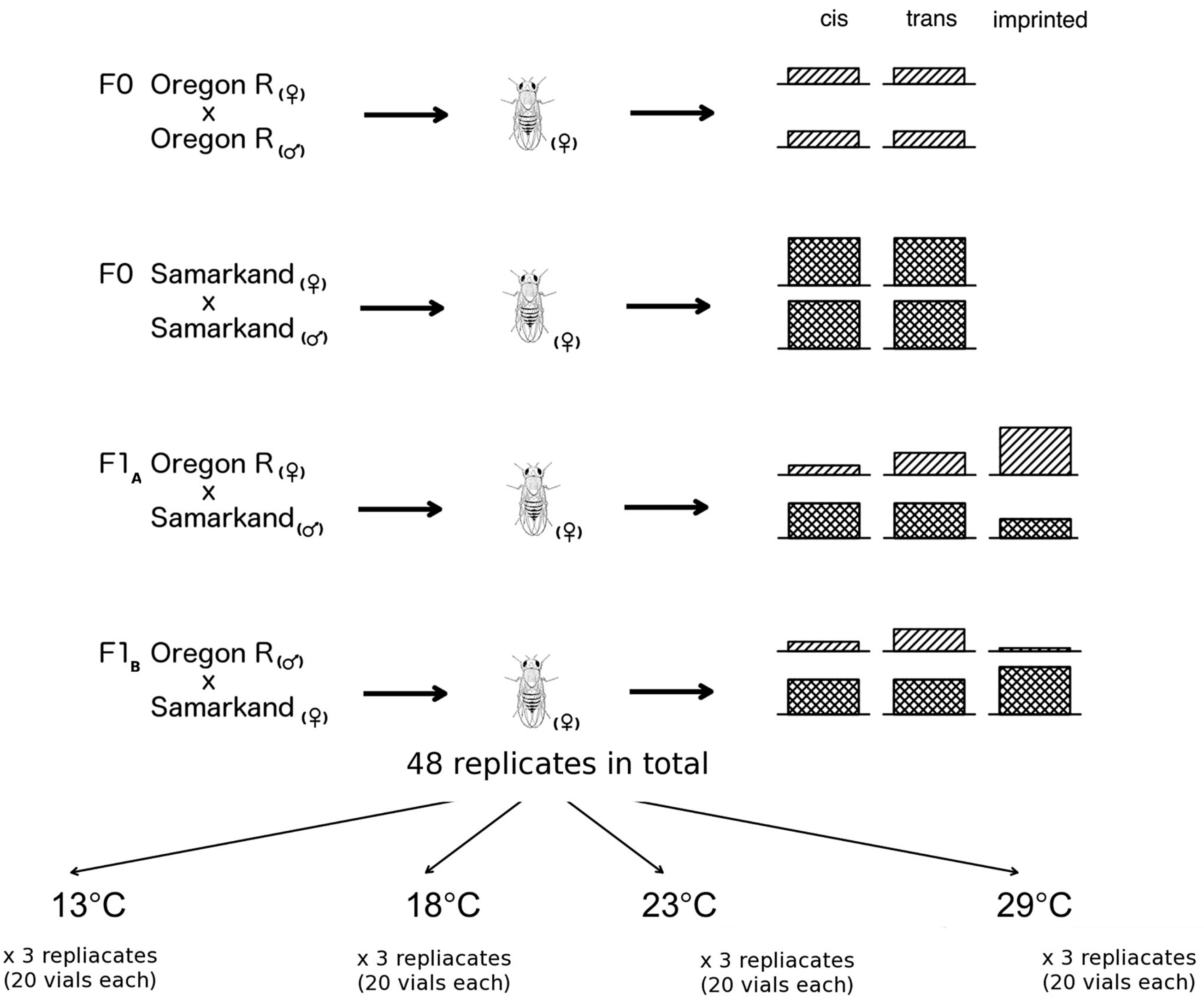

Fig 1. Overview of allele specific expression profiling by RNA-Seq in this study. RNA-Seq libraries were prepared from adult females of four different *D. melanogaster* crosses: two parental inbreds Oregon R and Samarkand, and two offspring crosses in both parent-of-origins ($F1_A$ and $F1_B$). Three replicates for each cross was reared in four different developmental temperatures and in total 48 replicates were collected in this study.

doi:10.1371/journal.pgen.1004883.g001

contrast, for flies either kept at 13°C or 23°C the number of differentially expressed genes increased to more than 1,000 (15%). The largest difference between the parental strains was observed at 29°C, where 2,581 genes (32.9%) were differentially expressed. The same trend of canalization/decanalization was seen when we compared the absolute expression difference between Oregon R and Samarkand strains across all expressed genes, with flies at 18°C exhibiting the lowest absolute $\log_2$ fold-difference (mean = 0.47, Fig. 2), followed by flies at 23°C (mean = 0.50, Fig. 2). Flies maintained at 13°C (mean = 0.54, Fig. 2) and 29°C (mean = 0.59, Fig. 2) had the highest difference in gene expression (all pairwise differences were highly significant; Wilcoxon test, FDR<0.01,).

To eliminate a potential bias in gene expression differences caused by variation in sequencing depth, we down-sampled the data sets to similar read counts by randomly picking paired-end reads without replacement. The final number of reads was determined ranking for each temperature the replicates by the number of reads. For each rank the number of reads was

Table 1. Summary of allele specific mapping.

	Temperature	13°C	18°C	23°C	29°C
**Oregon R**	#raw reads	35,439,037	38,040,969	37,245,502	42,845,478
	#reads mapped[a]	7,029,863	8,619,841	8,291,619	10,621,740
	#reads mapped to features[b]	6,469,806	8,072,438	7,791,375	9,985,236
**Samarkand**	#raw reads	34,443,487	39,811,213	38,976,587	50,131,346
	#reads mapped	6,180,263	7,409,968	8,734,436	12,121,409
	#reads mapped to features	5,553,249	6,705,893	8,115,453	11,295,071
**$F1_A$**	#raw reads	43,672,116	40,889,194	41,640,367	45,117,839
	#reads mapped	7,612,730	8,117,870	8,673,045	10,400,050
	#reads mapped to oregonR (%)[c]	51.4	51.7	51.3	51.2
	#reads mapped to features	6,945,409	7,483,157	8,063,002	9,661,667
**$F1_B$**	#raw reads	42,796,478	42,474,298	42,725,914	43,042,101
	#reads mapped	7,721,481	8,532,061	9,036,195	10,166,985
	#reads mapped to oregonR (%)[c]	48.4	47.9	48.6	49.2
	#reads mapped to features	7,036,089	7,784,495	8,381,386	9,471,969

Mean number of read pairs mapped across three replicates are given for in parents and F1 at four temperatures.

a: number of read pairs unambiguously mapped to concordant parental genome

b: number of read pairs mapped to unambiguous regions of *D. melanogaster* annotation feature

c: We noticed a slight imbalance between $F1_A$ and $F1_B$ for the number of reads mapped to the Oregon R reference. This imbalance is due to mitochondrial gene expression, which approximates about 1% of all reads. Since, $F1_A$ has the mtDNA genome from Oregon R, more reads map to the Oregon R reference.

doi:10.1371/journal.pgen.1004883.t001

matched to the one with the smallest number of reads. This procedure maximized the number of reads to be included in the matched samples (S1 Table). Importantly, we found the same trends in the down sampled data (S2 Table). Additionally, to rule out that heterogeneous read coverage across the whole gene body could affect our results, we summarized the gene expression differences using only reads aligned to the 500bp at the 3'end. Again, the same trends remain (S3 Table).

Table 2. Summary of genes with expression differences.

		13°C	18°C	23°C	29°C
**Divergence in F0**		1016	92	1210	2581
**Allelic expression divergence**					
	Ambiguous (ambig)	2375	1246	1248	1789
	Not different (n.s.)	3717	5855	4911	3369
	Compensatory	281	18	86	104
	cis × *trans*	18	0	13	31
	cis + *trans*	144	15	75	77
	trans only	501	17	485	1586
	cis only	153	38	371	233
**Inheritance modes of gene expression**	Not different (n.s.)	3470	7162	6375	4878
	O-dominant (O-dom)	153	11	200	2230
	S-dominant (S-dom)	3287	16	553	52
	Additive	71	0	48	17
	Over- dominant	85	0	13	7
	Under-dominant	123	0	0	5

doi:10.1371/journal.pgen.1004883.t002

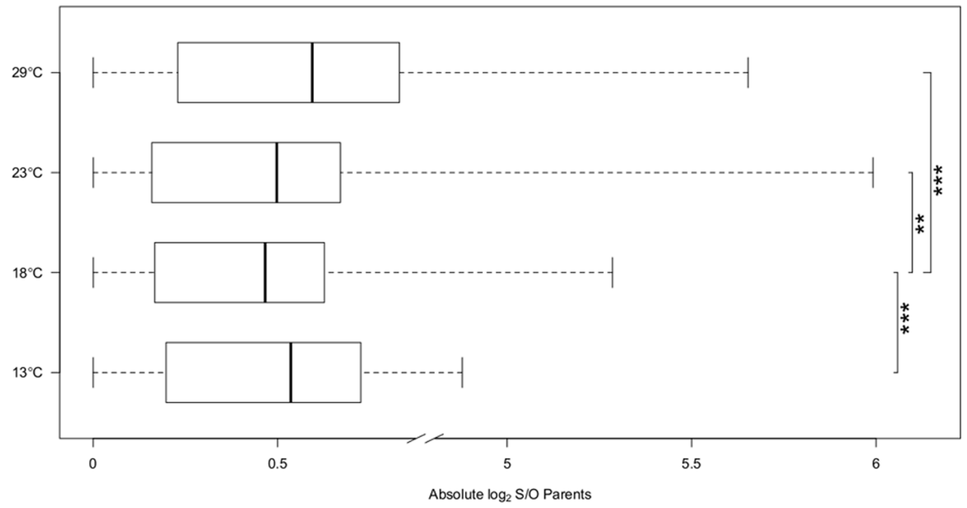

Fig 2. Expression divergence between Oregon R (O) and Samarkand (S). The box plots of the absolute $\log_2$ expression ratio in Samarkand and Oregon R parents across different temperatures. At 18°C the expression pattern between the two strains is most similar, while at the most extreme temperatures the expression is most diverged, suggesting temperature associated decanalization of gene expression. *P*-values are present for pairwise comparisons between 18°C and three other temperatures ('**'<0.01, '***' <0.001).

doi:10.1371/journal.pgen.1004883.g002

Since we detected in three libraries (t29-rep2 OregonR, t23-rep2 Samarkand, and t18-rep3 F1A) expression of male specific genes we suspected that a small fraction of males were inadvertently included in the flies used for RNA extraction. In order to rule out that any of the conclusions drawn in this manuscript we removed the contaminated samples as well as two other libraries of similar sizes to balance the replicate size in that temperature (see S1 Table). In addition, we used only reads mapping to the 500bp at the 3' end. The analysis using this reduced data set we confirmed the same patterns we saw for the full data set, albeit less pronounced due to the reduced power (S4 Table).

Decanalization of cis- and trans-regulation

Next, to dissect the regulatory architecture underlying these temperature specific effects, we measured allele specific gene expression by comparing expression of Oregon R and Samarkand alleles in both parents and offspring (Fig. 3). At 18°C, only a small fraction of genes had significant *trans*-regulatory (50, 0.7%) and *cis*-regulatory (71, 1.0%) effects. Flies maintained at 23°C, had approximately 1,000 genes with allelic regulatory divergence: 659 (9.2%) with *trans*-regulatory effects and 545 (7.6%) with *cis*-regulatory effects. For the two most extreme temperatures 944 (13.1%) genes differed in allelic expression due to *trans*-acting variants at 13°C and 1,798 (25.0%) at 29°C. The number of genes affected by *cis*-regulatory variants was 596 (8.3%) at 13°C, and 445 (6.19%) at 29°C (Table 2). Importantly, these results were not affected by different library sizes, since we obtained similar results for a down sampled data set (S2–S3 Tables). Thus, consistent with previous results [28] we found that temperature stress affects both *cis*- and *trans*-regulatory variants, with *trans*-effects being more common. When we compared the effect sizes by correlating *cis*- and *trans*-effects across temperatures, we found a striking difference. While *cis*-effects changed moderately with temperature difference (mean Spearman's $r = 0.86$, Fig. 4a), *trans*-effects were practically uncorrelated at different environmental temperatures (mean $r = 0.14$, Fig. 4a, c). Previous studies contrasting different yeast strains have identified a similar pattern of pronounced

trans-effects under stressful conditions [23,29]. In our study *cis*-regulated genes are also condition-dependent. Among the genes with temperature-specific *cis*-effects a considerable fraction did not show any *cis*-effects at any other temperature (13°C: 43%, 23°C: 24%, 29°C: 16%, Fig. 4b).

Until now, we analyzed each temperature separately, hence we complemented this analysis estimating allelic difference, temperature, and their interaction jointly. Consistent with our other analysis this approach identified at least 1200 genes differing in expression level between F0 strains across four temperatures (S4 Table). We also identified 480 genes were regulated by *cis*-effect across all temperatures while the number of trans-effects was close to 4,994. We found that 22 *cis*-effects and 4,300 *trans*-effects had significant interactions with temperature. This confirms that both regulatory effects are condition-dependent, and that *trans*-effects are more pronounced. For about 5,000 genes the expression level changed significantly across temperatures in either F0 or F1 datasets. 3,336 of them were found in common as temperature response genes in all tests (a detail gene list can be found in S1 Dataset).

Dominance of gene expression

To test the influence of the large number of *trans*-effects, we determined the mode of inheritance (i.e.: degree of dominance) by comparing the total expression levels of offspring to that of both parents (S3 Fig.). Most genes showed either no difference or were dominant, and only few

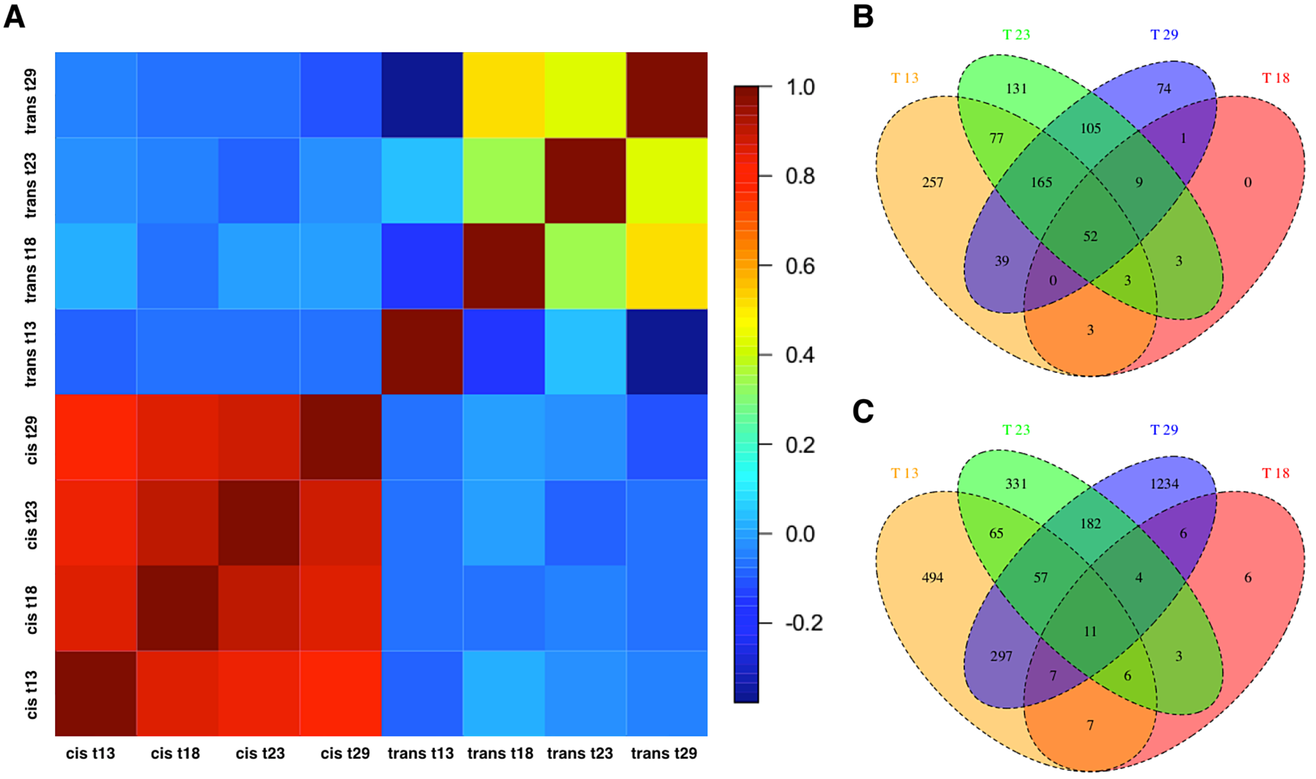

Fig 3. Temperature dependence of *cis*- and *trans*-regulatory differences. Scatter plots contrasting the relative allelic expression levels of parents and F1 offspring. Since the large number of genes makes a quantitative assessment difficult, we also show the density distribution for each class of genes. For representation purposes density distribution of genes with no significant differences in gene expression (yellow) is scaled by 1/10. While at (B) 18°C almost no allelic heterogeneity is present, the number of *cis*- and *trans*-effects increases with more extreme temperatures, (A) 13°C (C) 23°C and (D) 29°C.

doi:10.1371/journal.pgen.1004883.g003

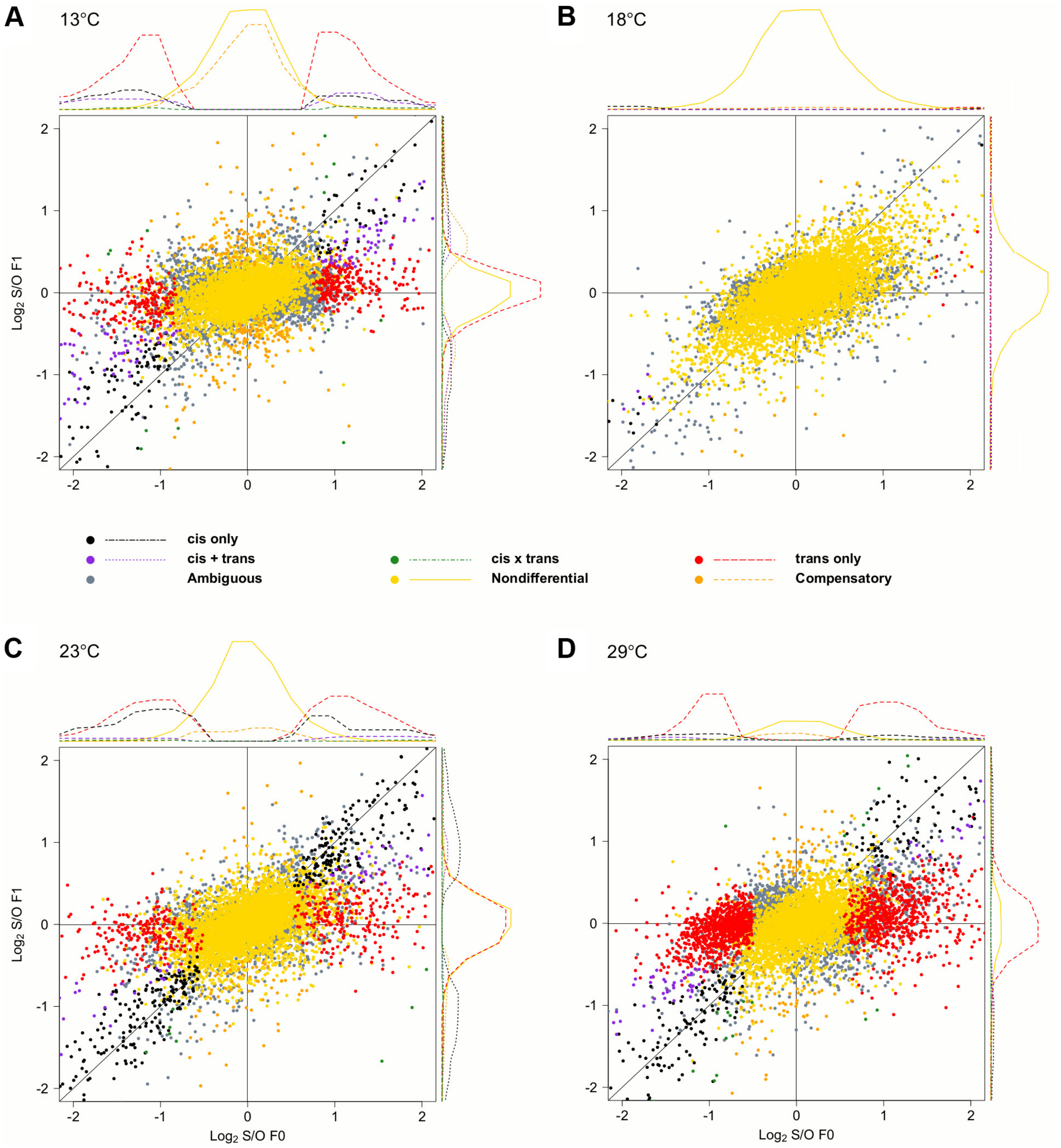

Fig 4. Temperature-dependence of *cis*- and *trans*-effects. (a) Pairwise correlation coefficient matrix (Spearman's *r*) between *cis*-effects and *trans*-effects across all temperatures. The correlation of *cis*-effects across environments was more similar than the one of trans-effects. (b) Venn Diagram showing the number of *cis*-regulated and (c) *trans*-regulated genes at four different temperatures.

doi:10.1371/journal.pgen.1004883.g004

genes (<3.9%) were classified as being additive, under-dominant, and over-dominant (Fig. 5). Again, we noticed a very striking dependence on temperature, with flies at 18°C showing the least differences between parents and offspring. Only 27 genes showed dominance in gene expression. At 23°C, 200 genes (2.8%) were Oregon R-dominant, and 553 (7.7%) were Samarkand-dominant. At the two extreme temperatures, up to 51.7% of the expressed genes showed

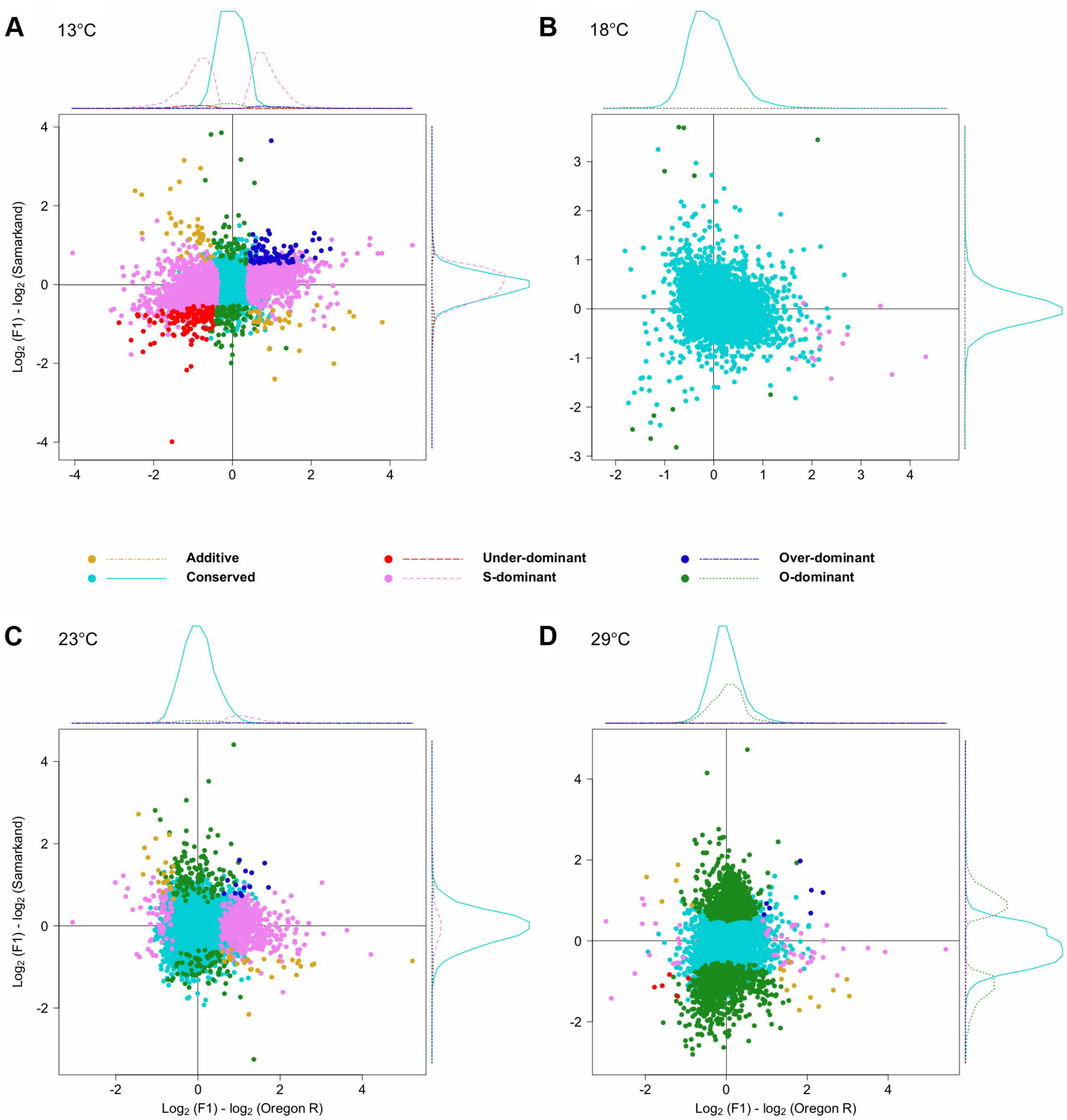

Fig 5. Temperature dependent inheritances of gene expression levels. Scatter plots summarizing the inheritance patterns by contrasting the differences of each of the parental lines to the F1. Since the large number of genes makes a quantitative assessment difficult, we also show the density distribution for each class of genes. . For representation purposes density distribution of genes with no significant differences in gene expression (light blue) is scaled by 1/10. Again, at (B) 18°C the fewest differences were noted compared to (A) 13°C, (C) 23°C, and (D) 29°C.

doi:10.1371/journal.pgen.1004883.g005

dominance, but the distribution was not balanced between the two strains (Table 2). While at 13°C the majority of dominant alleles (3,287, 45.7%) had an expression level resembled the Samarkand parent with non-significant or negligible change (<1.25-fold), at 29°C the majority (2,230, 31%) resembled the Oregon R parent. Among the genes with dominant gene expression, only a moderate number showed allelic imbalance: 504 (14.6% of dominant genes) at 13°C, 19 (70.4%) at 18°C, 267 (35.5%) at 23°C, and 244 (10.7%) 29°C, suggesting that for the majority of genes both parental alleles are subject to the same regulatory input.

"Dominance-swapped" genes are enriched for a few transcription factor binding sites

Among the genes with a dominant expression pattern at either 13°C or 29°C, 1,384 genes were dominant in both environments, but their dominance pattern was reverted between the two temperatures (hereafter we refer to these genes as "dominance-swapped" genes). Since among these swapped genes the fraction of *trans*-regulated ones is very high (up to 66%), we reasoned that they may be regulated by a few transcription factors (TFs). We therefore performed an *in silico* analysis of enrichment for transcription factor binding sites among these dominance-swapped genes. The binding sites of 13 TFs were significantly overrepresented (S5 Table). We further investigated whether a combination of TFs may be enriched, rather than a single one. Indeed, we found that several combinations of TFs may affect the expression of dominance-swapped genes. The most highly ranked combination of *Chro* and *BEAF-32* may affect the expression of up to 934 genes (68% of the dominance swapped genes), while the largest combination contained 11 of the 13 TFs with binding site enrichment in the single TF analysis (S6 Table).

Eight out of these 13 TFs were present in our data set (Table 3). Among these, three TFs showed dominance and two of them also exhibited swapped dominance. Three TFs had *cis*- (*mip120*) or *trans*-effects (*Med*, *BEAF-32*, and *mip120*). Of particular interest is the transcription factor *mip120*, which is affected by *cis*-regulatory variants at 29°C and at 13°C it is regulated by both *trans*- and *cis*-regulatory variants. It is possible that the *cis*-regulatory variants are responsible for *trans*-regulatory divergence of multiple downstream TFs, causing the coordinated expression of hundred of genes (i.e. sensory *trans* factors *sensu* [23]).

To shed more light onto the biology of the dominance-swapped genes, we performed Gene Ontology (GO) and pathway analyses and found many genes involved in macromolecule biosynthesis, especially mRNA translation to be overrepresented (S7 Table). Gene ontology categories for cellular components were enriched for mitochondrial ribosome and vitelline membrane. Other molecular functions and bioprocesses had also been identified, annotated as neural signal transmitting, transmembrane transporter activity, and body fluid secretion. On

Table 3. Allele specific expression and inheritance patterns for transcriptional factors enriched in dominance swapped genes.

	Genes	13°C		18°C		23°C		29°C	
**Transcription factors**		Allelic	Inherit	Allelic	Inherit	Allelic	Inherit	Allelic	Inherit
	mip120	***cistrans***	**S-dom**	n.s.	n.s.	ambig.	n.s.	***cis***	**O-dom**
	Med	n.s.	n.s.	n.s.	n.s.	ambig.	n.s.	***trans***	**O-dom**
	BEAF-32	n.s.	**S-dom**	n.s.	n.s.	n.s.	n.s.	***trans***	**O-dom**
	trx	n.s.	**S-dom**	n.s.	n.s.	ambig.	n.s.	n.s.	n.s.
	dl	ambig.	**S-dom**	n.s.	n.s.	n.s.	n.s.	n.s.	n.s.
	CtBP	n.s.	n.s.	n.s.	n.s.	ambig.	n.s.	ambig.	n.s.
	phol	n.s.	n.s.	n.s.	n.s.	n.s.	n.s.	n.s.	n.s.
	Chro	n.s.	n.s.	n.s.	n.s.	n.s.	n.s.	n.s.	n.s.

doi:10.1371/journal.pgen.1004883.t003

the other hand, genes with *cis*- and *trans*-regulatory effects are not enriched for any GO categories or pathways (after removing genes common with dominance-swapped genes).

Discussion

Analyzing allelic effects on gene expression for flies grown at four different developmental temperatures we found the striking pattern of a large number of *trans*-effects at stressful temperatures. Similar results were obtained for yeast where stressful culture conditions resulted in large *trans*-effects [23,29] and *C. elegans* where 59% of the *trans*-acting genes showed a significant eQTL by environment interaction [28]. The important difference of our study to the previous ones is that we tested multiple temperatures covering stressful and less stressful conditions. Since for flies grown at 18°C the genetic differences between the two strains had almost no influence on the levels of gene expression, our data provide experimental evidence for canalization of gene expression. At stressful conditions, this canalized gene expression is disturbed resulting in many significant differences between the two parental strains as well as significant *cis*-and *trans*-effects.

Following the widely accepted hypothesis that canalization of traits is the consequence of stabilizing selection (reviewed in [3,4]), our data suggest that the most benign temperature for *D. melanogaster* is 18°C. A similar line of argument has as been applied to the evolution of reaction norms. For fitness related traits, such as egg production, it has been proposed that the maximum egg production should be observed for the optimal temperature. While our experiment suggested an optimal temperature of 18°C, the optimal temperature for ovariole number [30] and fecundity [31] is closer to 23°C than to 18°C. It is thus not clear if the reaction norm of fitness traits could serve as good indicator for optimal temperatures. On the one hand the reaction norms tend to be very similar for flies collected from different environments [30,31], while on the other hand the optimal temperature seems to differ among reaction norms. Finally, thermotactic studies demonstrated that 18°C is the preferred temperature of *D. melanogaster* [32,33], suggesting that flies prefer temperatures with canalized gene expression.

Our study has relied on two old laboratory strains that have been established more than 80 years ago [34], and since that time they have probably largely been maintained at 18°C to reduce the number of transfers. We can therefore not rule out that canalization might have evolved to match these common laboratory culture conditions. Nevertheless, comparison of various *D. melanogaster* strains at 25°C identified several hundred differentially expressed genes [35]. Similarly, a study based on the DSPR lines detected 7922 eQTLs at 25°C [36], demonstrating considerable allelic effects in gene expression. While a proper comparison would require an analysis of gene expression at different temperatures, the substantial number of significant differences in gene expression among genotypes at 25°C suggests that gene expression was also decanalized in these studies. Thus, our observation of strongly canalized gene expression at 18°C may not be specific to the strains used in our study.

By uncovering otherwise hidden genetic variation decanalization could facilitate adaptation of populations exposed to a novel, stressful environment. On the other hand, many of the otherwise cryptic variants are expected to be deleterious as recently pointed out by Gibson et al. [37]: environmental and/or genetic stress associated with the recent history of human populations might have resulted in decanalization and a consequence in an increase of complex human genetic disorders. General stressors, such as smoking, have been shown to be a contributing factor to many complex diseases [38]. While it is not proven that decanalized gene expression is causative for these diseases, it is remarkable that for some genetic disorders allelic imbalance in gene expression has been observed [39–42]. Our results considerably strengthen the notion that decanalization might commonly manifest itself as a major allelic imbalance in gene expression.

Like most studies on canalization [5,43,44], our analysis was based on naturally occurring variants. Since, purifying selection is removing deleterious variation and directional selection increases the frequency of favored variants, it may be possible that natural selection could influence our interpretation of canalization. Assuming that regulatory variants are temperature specific and purifying selection is more effective at 18°C, it may be possible that variation influencing gene regulation at 18°C is purged from the population, while the other variants remain segregating in the population. Alternatively, new mutations occurring during the propagation of the classic laboratory strains may have been purged more efficiently at 18°C since laboratory strains are typically maintained at this temperature, albeit at very small population sizes. The outcome of such scenarios would be indistinguishable from the pattern seen in our study. Consistent with the idea of natural selection mimicking the effect of canalization a recent study failed to verify the previously described effect of the histone variant HTZ1 on mutational robustness in mutation accumulation lines for which the effect of natural selection is minimized [45]. Our current understanding of the regulation of gene expression is not sufficiently advanced to decide if sequence variants are affecting gene expression in a temperature specific manner such that selection could remove variants specific to 18°C but other variants could accumulate in natural populations. Future experiments employing mutation accumulation lines or experimental evolution will be instrumental to distinguish between the two scenarios.

The phenomenon of temperature dependent dominance in gene expression seen in our experiments is particularly interesting. A high frequency of genes with dominant modes of inheritance in gene expression has already been documented in other studies in Drosophila [24,46], yeast [47] and *A. thaliana* [48]. The novelty of our study is, however, that for a large fraction of genes this mode of inheritance depends on temperature: genes with dominance of the Samarkand allele are showing dominance of the Oregon R allele at the other temperature extreme (Table 2). Our *in silico* analysis suggested that many genes with such a pattern of swapped dominance are potentially regulated by a few transcription factors, of which two also exhibit a temperature dependent swap in the mode of inheritance (Table 3). While it is conceivable that these transcription factors are part of a regulatory network of the swapped genes, the question of the regulation of one or a few master regulators deserves special attention. One simple way to achieve dominance in gene expression is loss of function of one of the parental alleles. This model, however, seems not to apply to our data since the genes are expressed in both parental strains. Alternatively, the dominance may be the result of a different tissue representation in the two parental strains. It has been described that ovary sizes/ ovariole number differ among *D. melanogaster* strains [e.g.: 49]. If the F1 does not have an intermediate ovary size but are similar to one of the parents, genes predominantly expressed in the ovaries are expected to show dominance. We tested the hypothesis of differential ovary sizes by comparing the expression of chorion genes and did not find support for this hypothesis since they largely showed no dominance pattern (S8 Table). We also excluded that the swapping dominance is the outcome of a temperature × parental origin interaction (688 swapped dominance genes in $F1_A$ and 979 in $F1_B$, S9 Table). One interesting observation in *C. elegans* linked dominance in gene expression to nonsense mediated mRNA decay (NMD) [50]: in NMD impaired individuals many mutations were highly dominant, while with a functional NMD the same mutations were recessive. While it is conceivable that NMD activity may be affected by temperature, it is not clear how the swapped dominance could be caused. Finally, dominance may be the outcome of some form of allelic crosstalk, either by transvection [51–53] or other mechanisms [54]. How environmental signals are incorporated in such a mechanism, remains an open question.

Materials and Methods

Animal rearing and handling

Flies were reared on standard cornmeal-molasse-yeast-agar medium and maintained at 12 h light/12 h dark conditions. Prior to crossing, the parental strains were subjected to 7 generations of sibling pair matings in order to reduce residual heterozygosity. Virgin females of either strain were used for the following four crosses: O female × O male, and S female × S male (F0 parents); O female × S male (hybrid cross $F1_A$), and S female × O male (hybrid cross $F1_B$). For each type of crosses, three replicates were set up in parallel, each consisting of approximately 80 crosses of a single female and a single male. After two days of egg laying at 23°C four subsets of 20 vials was transferred at four different temperatures (13°C, 18°C, 23°C, and 29°C).

Sample preparation and sequencing

Virgin females were collected from both parents and F1 flies shortly after eclosion and aged three days before shock-freezing in liquid nitrogen. For each replicate (out of a total of 48), approximate 30 females were homogenized in peqGOLD TriFast Reagent (Peqlab, Erlangen, Germany) using an Ultraturrax T10 (IKA-Werke, Staufen, Germany). Total RNA was extracted, quality-checked on agarose gels, and quantified using the Qubit RNA Assay Kit (Invitrogen, Carlsbad, CA, USA). Paired-end Illumina mRNA libraries were generated from 5μg total RNA. After DNase I treatment (Qiagen, Hilden, Germany), poly(A) transcripts were isolated using the NEBNext Poly(A) mRNA Magnetic Isolation Module (New England Biolabs, Ipswich, MA). Strand-specific paired-end libraries were prepared using the NEBNext Ultra Directional RNA Library Prep Kit for Illumina and size-selected on AMPureXP beads (Beckman Coulter, CA, USA) aiming for fragments between 380–500bp. All libraries were amplified with 12 PCR cycles using index primers from the NEBNext Multiplex Oligos for Illumina Kit (New England Biolabs, Ipswich, MA) and sequenced on a HiSeq2000 using a 2×100bp protocol. Our barcoding scheme was such that all 16 samples of a single replicate were included in the same lane(s) to minimize lane effects.

Allele specific gene expression

S1 Fig. illustrates the whole allele specific mapping procedure and statistic analyses applied in this study. We first trimmed all sequence reads using the Mott algorithm implemented in PoPoolation [55] and aligned reads of the two parents separately to the Flybase *D. melanogaster* 5.49 assembly. We identified substitutions in the two parental strains relative to the reference genome using variants occurring at a frequency $\geq$ 0.98 with a read-depth $\geq$2. Those variants were used to perform a second round SNP-tolerant read alignment using GSNAP [56,57] in order to get the final parental specific SNP datasets with higher confidence. A total number of 177,107 and 193,699 SNPs (i.e.: variants relative to the reference genome) were identified in Oregon R and Samarkand respectively. Amongst those, 182,123 SNPs differed between two parents. The read depths of SNPs were high correlated between two parental data sets (Pearson's cor. coef = 0.94, *p*-value <2.2e-16, S2 Fig.), suggesting the SNP discovery had suffered marginal bias towards either of parents. We generated two parental specific genomes by modifying the *D. melanogaster* reference using Oregon R and Samarkand SNP datasets.

Reads were assigned to one of the parental genotypes by aligning them simultaneously to both parental genomes. Only unambiguously mapped reads in proper pairs were assigned to either O or S. Reads mapping to both genomes equally well were not included in the analyses. Allelic expression was measured using the ReCOG software tool (https://code.google.com/p/recog/) on each of the two reference genomes separately. We only counted reads that were mapped fully within the gene boundaries. The only exceptions were overlapping genes. Here,

the overlapping region was considered ambiguous and not counted. On average 21% of the reads could be assigned to one parental reference genome. Using the RNA-Seq reads from the parents we discovered that 0.19% and 0.27% of the reads were assigned to the wrong parental genome. To account for these potential mapping errors, we followed a previously suggested strategy [58] and simulated about 18,000,000 paired-end reads from both parental genomes and assigned them to either of the reference genomes using the same strategy as for the real data. Equal expression levels for both alleles were expected in the simulated data for all genes, given no mapping errors. Therefore, we excluded 15 genes with simulated allelic expression divergence to avoid biased divergence estimation caused by mapping errors. The protocols described above are implemented in the package ALLIM [58].

Finally we filtered for a minimum expression level using the following criteria. A gene is defined as expressed in the F0 flies if in all samples at least one read was mapped and at least one sample had ≥20 counts. In F1 flies the expression counts to both reference genomes were considered jointly and the same cutoffs as for the parental samples was applied. For a gene to be considered expressed in all samples, the criteria for F0 and F1 flies had to be met. Out of 18,764 *D. melanogaster* genes / features annotated in either Flybase r5.49 [59] or the developmental transcriptome [60], we detected the expression of 7,853 (41.8%) genes in F0 flies, 7,208 (38.4%) genes in F1 flies, and 7,191 (38.3%) genes in both F0 and F1 flies.

Identifying imprinted genes

We used the F1 crosses that were carried out in both directions to identify imprinted genes. Read counts of each gene were normalized by total library size and RNA composition of each data set using a trimmed mean of M-values method [61]. For each gene, a generalized linear model (GLM) was applied to evaluate the divergence of allelic expression levels between the F1 crosses in two parent-of-origin orders ($F1_A$ and $F1_B$), at four temperatures separately:

$$log_2(\frac{O}{O+S})_{F1} \sim \begin{pmatrix} F1_A \\ F1_B \end{pmatrix} + \varepsilon$$

where ε denotes the error term and the quasi-binominal distribution was used to account for the over-dispersion. *P*-values were calculated by F-test followed by FDR correction. Two putative imprinted genes were excluded from our further analyses of expression divergence, which resulted in a total number of 7,189 genes expressed in F0 and F1.

Cis- and trans-regulatory divergence assignment

The parental (F0) data sets were first tested for significance of differential gene expression, and offspring (F1) were tested for differential allelic expression at each temperature separately. We applied TMM normalization on read counts of each gene and performed an empirical Bayesian estimation based on negative-binomial GLM to compute gene-wise dispersions [62,63]. The significance of expression divergence was determined by an F-test:

$$Allelic\ Expression \sim \begin{pmatrix} allele\ oregonR \\ allele\ Samarkand \end{pmatrix} + \varepsilon$$

We further compared the strain-specific allele abundance ratio between F0 and F1 data sets:

$$log_2(\frac{O}{O+S}) \sim \begin{pmatrix} F0 \\ F1 \end{pmatrix} + \varepsilon$$

Quasi-binominal GLM analysis was performed for each gene and any significant difference between F0 and F1 data set was considered as evidence of *trans* effects (T).

For all statistical analyses applied in F0, F1 and T, *p*-values were adjusted by FDR correction [64] with a nominal cutoff of $\leq$ 5%. Genes were classified into seven categories by comparing the significance levels from all three tests [24,47]:

1. *Not different*: No significant differential expression in F0 or F1. No significant T.
2. *Cis* only: Significant differential expression in F0 and F1. No significant T.
3. *Trans* only: Significant differential expression in F0, but not in F1. Significant T.
4. *Cis* + *trans*: Significant differential expression in F0 and F1. Significant T. *Cis*- and *trans*-regulatory effects favor expression of the same allele.
5. *Cis* × *trans*: Significant differential expression in F0 and F1. Significant T. *Cis*- and *trans*-regulatory effects favor expression of the different allele.
6. *Compensatory*: Significant differential expression in F1, but not in F0. Significant T. Expression difference caused by *cis*- and *trans*-regulatory components had an opposite direction and perfectly compensate each other such that no expression difference in F0.
7. *Ambiguous*: Significant in only one of differential expression tests in F0, F1 or T. Thus, no explicit *cis-*/*trans*-effect can be detected.To further confirm our estimates of gene/allelic expression difference, we made a joint estimation using GLM method including allelic difference, temperature, and their interaction:

$$Allelic\ Expression \sim \begin{pmatrix} allele\ oregonR \\ allele\ Samarkand \end{pmatrix} \times temperature + \varepsilon$$

or

$$log_2\left(\frac{O}{O+S}\right) \sim \begin{pmatrix} F0 \\ F1 \end{pmatrix} \times temperature + \varepsilon$$

Inbred-hybrid divergence assignment

We evaluated the divergence of total expression (i.e.: ignoring allelic information) between F0 parents and F1 hybrids for each gene and for each temperature, following the previously suggested "mode of inheritance classification" [24,47]. The total gene expression level in F1 flies was estimated as the sum of reads mapped to both parental alleles. TMM normalization followed by a negative-binominal GLM analysis was used to evaluate the expression values of F1 flies between either of the two parents (F0):

$$Total\ Expression \sim \begin{pmatrix} F0_{oregonR} \\ F1 \end{pmatrix} + \varepsilon$$

or

$$Total\ Expression \sim \begin{pmatrix} F0_{samarkand} \\ F1 \end{pmatrix} + \varepsilon$$

Genes that have a parent / offspring expression ratio over 1.25-fold and an adjusted *p*-value

≤5% were considered as diverged between F0 and F1 samples and were classified as additive, Oregon R-dominant, Samarkand-dominant, under-dominant, or over-dominant inheritance, based on the magnitude of the difference between total expression in the F1 and in each parental sample (S3 Fig.).

Enrichment test for transcription-factors and gene sets

Transcription factor (TF) enrichment was tested by comparing the number of target genes for each TF between a test gene-set and all expressed genes. Significance levels were determined by a one tailed hyper-geometric test. We estimated the number of false positives by 1000 random samples, with each sample consisting of the same number of genes as in the test set. The association of multiple transcription factors was investigated by the Limitless-Arity multiple-testing procedure [65]. The significance was calculated using Mann-Whitney U-test and was calibrated by Family-Wise Error Rate. We performed these transcription factor enrichment tests on 149 experimentally verified transcription factors collected from the Drosophila Interactions Database version 2013–07 [66]. The number of regulatory transcription factors in different test sets was used to compare with those in all expressed genes.

Gene-set enrichment analysis was carried out with the software FUNC [67], using all expressed genes as a background gene list. The pathway analysis was performed with the R package "gage" [68]. Genes were mapped to KEGG pathways and pathways enriched with genes of expression divergence were reported.

We have deposited all RNA-Seq raw sequencing reads in NCBI Sequence Read Archive with accession numbers SRP041398 (F0) and SRP041395 (F1). All read-count tables and customized R scripts for statistical analyses have been uploaded to DataDryad.org with accession doi: 10.5061/dryad.pk045.

Supporting Information

S1 Fig. Overview of allele specific expression inference procedure.
(TIFF)

S2 Fig. The correlation of SNP coverage between two parental data sets. The high correlation suggests that no mapping bias exists.
(TIFF)

S3 Fig. Barplots illustrating the six inheritance modes as determined by the comparison of F1 samples to the parental samples.
(TIFF)

S4 Fig. Test for imprinted genes. The high correlation of the allelic expression profile between $F1_A$ and $F1_B$ suggests genomic imprinting is absent in D. melanogaster adult female flies. Only for two genes we detected significant imprinting between $F1_A$ and $F1_B$. ChrU_5299041_5299681.0, which is located on the mtDNA, exhibited a dramatic expression change (indicated by the star symbol), while only minor expression change was detected for CG1275 (indicated by the dot near origin).
(TIFF)

S1 Table. Number of read pairs mapped to parental alleles after down-sampling.
(DOCX)

S2 Table. Summary of genes with expression differences after down-sampling.
(DOCX)

S3 Table. Summary of expression differences using reads aligned to 500bp at the 3'end of genes after down-sampling.
(DOCX)

S4 Table. Summary of joint estimated effects of allelic difference, temperature and their interaction on gene expression.
(DOCX)

S5 Table. Enrichment tests of transcription factors in dominance-swapped genes against all expressed genes.
(DOCX)

S6 Table. Enrichment tests of combinations of multiple transcription factors in dominance-swapped genes (only top 100 combinations shown here).
(DOCX)

S7 Table. Top ranked GO terms and core pathway sets from gene set enrichment analyses performed on dominance-swapped genes.
(DOCX)

S8 Table. Inheritance of gene expression pattern in chorion protein genes.
(DOCX)

S9 Table. Inheritance mode of gene expression in F1 with two parent-of-origin orders.
(DOCX)

S10 Table. Inheritance mode of gene expression in F1 with two parent-of-origin orders.
(DOCX)

S1 Dataset. Detail list of genes in expression differentiation based on joint estimation of allelic difference, temperature and interactions.
(XLSX)

Acknowledgments

We are thankful to A. Suvorov and D. Martynow for expert fly work. G. Gibson and T. Flatt provided helpful comments on earlier versions of the manuscript. We are grateful to P. Wittkopp for insightful feedback on the possibility of decanalization of gene expression in hybrids. Three reviewers provided valuable feedback, which helped us to improve the manuscript.

Author Contributions

Conceived and designed the experiments: CS. Performed the experiments: JC VN. Analyzed the data: JC. Wrote the paper: CS JC VN.

References

1. Schmalhausen II (1949) Factors of Evolution: The Theory of Stabilizing Selection. Philadelphia: The Blankiston Company.
2. Waddington CH (1942) Canalization of development and the inheritance of acquired characters. Nature 150: 563–565.
3. Siegal ML, Bergman A (2002) Waddington's canalization revisited: developmental stability and evolution. Proc Natl Acad Sci U S A 99: 10528–10532. PMID: 12082173
4. Flatt T (2005) The evolutionary genetics of canalization. Q Rev Biol 80: 287–316. PMID: 16250465
5. Rutherford SL, Lindquist S (1998) Hsp90 as a capacitor for morphological evolution. Nature 396: 336–342. PMID: 9845070

6. Jarosz DF, Lindquist S (2010) Hsp90 and environmental stress transform the adaptive value of natural genetic variation. Science 330: 1820–1824. doi: 10.1126/science.1195487 PMID: 21205668
7. Rohner N, Jarosz DF, Kowalko JE, Yoshizawa M, Jeffery WR, et al. (2013) Cryptic variation in morphological evolution: HSP90 as a capacitor for loss of eyes in cavefish. Science 342: 1372–1375. doi: 10.1126/science.1240276 PMID: 24337296
8. Waddington CH (1953) Genetic assimilation of an acquired character. evolution 7: 118–126.
9. Waddington CH (1956) Genetic assimilation of the bithorax phenotype. evolution 10: 1–13.
10. Polaczyk PJ, Gasperini R, Gibson G (1998) Naturally occurring genetic variation affects Drosophila photoreceptor determination. Development Genes and Evolution 207: 462–470. PMID: 9510541
11. Gibson G, Hogness DS (1996) Effect of polymorphism in the Drosophila regulatory gene Ultrabithorax on homeotic stability. Science 271: 200–203. PMID: 8539619
12. Gavin-Smyth J, Wang YC, Butler I, Ferguson EL (2013) A genetic network conferring canalization to a bistable patterning system in Drosophila. Current Biology 23: 2296–2302. doi: 10.1016/j.cub.2013.09.055 PMID: 24184102
13. Lott SE, Kreitman M, Palsson A, Alekseeva E, Ludwig MZ (2007) Canalization of segmentation and its evolution in Drosophila. Proc Natl Acad Sci U S A 104: 10926–10931. PMID: 17569783
14. Manu, Surkova S, Spirov AV, Gursky VV, Janssens H, et al. (2009) Canalization of gene expression in the Drosophila blastoderm by gap gene cross regulation. PLoS Biology 7: 591–603.
15. Marques-Pita M, Rocha LM (2013) Canalization and control in automata networks: body segmentation in *Drosophila melanogaster*. PLoS One 8.
16. Morris MR, Richard R, Leder EH, Barrett RD, Aubin-Horth N, et al. (2014) Gene expression plasticity evolves in response to colonization of freshwater lakes in threespine stickleback. Mol Ecol 23: 3226–3240. doi: 10.1111/mec.12820 PMID: 24889067
17. Levine MT, Eckert ML, Begun DJ (2011) Whole-genome expression plasticity across tropical and temperate *Drosophila melanogaster* populations from Eastern Australia. Mol Biol Evol 28: 249–256. doi: 10.1093/molbev/msq197 PMID: 20671040
18. Sikkink KL, Reynolds RM, Ituarte CM, Cresko WA, Phillips PC (2014) Rapid evolution of phenotypic plasticity and shifting thresholds of genetic assimilation in the nematode *Caenorhabditis remanei*. G3 (Bethesda) 4: 1103–1112. doi: 10.1534/g3.114.010553 PMID: 24727288
19. Runcie DE, Garfield DA, Babbitt CC, Wygoda JA, Mukherjee S, et al. (2012) Genetics of gene expression responses to temperature stress in a sea urchin gene network. Mol Ecol 21: 4547–4562. doi: 10.1111/j.1365-294X.2012.05717.x PMID: 22856327
20. Yampolsky LY, Glazko GV, Fry JD (2012) Evolution of gene expression and expression plasticity in long-term experimental populations of *Drosophila melanogaster* maintained under constant and variable ethanol stress. Mol Ecol 21: 4287–4299. doi: 10.1111/j.1365-294X.2012.05697.x PMID: 22774776
21. Goncalves A, Leigh-Brown S, Thybert D, Stefflova K, Turro E, et al. (2012) Extensive compensatory cis-trans regulation in the evolution of mouse gene expression. Genome Research 22: 2376–2384. doi: 10.1101/gr.142281.112 PMID: 22919075
22. Wittkopp PJ, Haerum BK, Clark AG (2004) Evolutionary changes in cis and trans gene regulation. Nature 430: 85–88. PMID: 15229602
23. Tirosh I, Reikhav S, Levy AA, Barkai N (2009) A yeast hybrid provides insight into the evolution of gene expression regulation. Science 324: 659–662. doi: 10.1126/science.1169766 PMID: 19407207
24. McManus CJ, Coolon JD, Duff MO, Eipper-Mains J, Graveley BR, et al. (2010) Regulatory divergence in Drosophila revealed by mRNA-seq. Genome Research 20: 816–825. doi: 10.1101/gr.102491.109 PMID: 20354124
25. Wittkopp PJ, Haerum BK, Clark AG (2006) Parent-of-origin effects on mRNA expression in *Drosophila melanogaster* not caused by genomic imprinting. Genetics 173: 1817–1821. PMID: 16702434
26. Menon DU, Meller VH (2010) Germ line imprinting in Drosophila: Epigenetics in search of function. Fly (Austin) 4: 48–52. PMID: 20081359
27. Coolon JD, Stevenson KR, McManus CJ, Graveley BR, Wittkopp PJ (2012) Genomic imprinting absent in *Drosophila melanogaster* adult females. Cell Reports 2: 69–75. doi: 10.1016/j.celrep.2012.06.013 PMID: 22840398
28. Li Y, Alvarez OA, Gutteling EW, Tijsterman M, Fu J, et al. (2006) Mapping determinants of gene expression plasticity by genetical genomics in *C*. *elegans*. PLoS Genet 2: e222. PMID: 17196041
29. Smith EN, Kruglyak L (2008) Gene-environment interaction in yeast gene expression. PLoS Biol 6: e83. doi: 10.1371/journal.pbio.0060083 PMID: 18416601

30. Delpuech JM, Moreteau B, Chiche J, Pla E, Vouidibio J, et al. (1995) Phenotypic plasticity and reaction norms in temperate and tropical populations of D*rosophila melanogaster* ovarian size and developmental temperature. evolution 49: 670–675.

31. Klepsatel P, Galikova M, De Maio N, Ricci S, Schlötterer C, et al. (2013) Reproductive and post-reproductive life history of wild-caught *Drosophila melanogaster* under laboratory conditions. Journal of Evolutionary Biology 26: 1508–1520. doi: 10.1111/jeb.12155 PMID: 23675912

32. Shen WL, Kwon Y, Adegbola AA, Luo JJ, Chess A, et al. (2011) Function of Rhodopsin in Temperature Discrimination in Drosophila. Science 331: 1333–1336. doi: 10.1126/science.1198904 PMID: 21393546

33. Kwon Y, Shim HS, Wang XY, Montell C (2008) Control of thermotactic behavior via coupling of a TRP channel to a phospholipase C signaling cascade. Nature Neuroscience 11: 871–873. doi: 10.1038/nn.2170 PMID: 18660806

34. Lindsley DL, Grell EH (1968) Genetic variations of *Drosophila melanogaster*. Washington, D.C.: Carnegie Institute of Washington Publication.

35. Jin W, Riley RM, Wolfinger RD, White KP, Passador-Gurgel G, et al. (2001) The contributions of sex, genotype and age to transcriptional variance in *Drosophila melanogaster*. Nat Genet 29: 389–395. PMID: 11726925

36. King EG, Sanderson BJ, McNeil CL, Long AD, Macdonald SJ (2014) Genetic dissection of the *Drosophila melanogaster* female head transcriptome reveals widespread allelic heterogeneity. PLoS Genet 10: e1004322. doi: 10.1371/journal.pgen.1004322 PMID: 24810915

37. Gibson G (2009) Decanalization and the origin of complex disease. Nature Reviews Genetics 10: 134–140. doi: 10.1038/nrg2502 PMID: 19119265

38. Ezzati M, Lopez AD (2003) Estimates of global mortality attributable to smoking in 2000. Lancet 362: 847–852. PMID: 13678970

39. Ge B, Pokholok DK, Kwan T, Grundberg E, Morcos L, et al. (2009) Global patterns of cis variation in human cells revealed by high-density allelic expression analysis. Nature Genetics 41: 1216–U1278. doi: 10.1038/ng.473 PMID: 19838192

40. Emilsson V, Thorleifsson G, Zhang B, Leonardson AS, Zink F, et al. (2008) Genetics of gene expression and its effect on disease. Nature 452: 423–U422. doi: 10.1038/nature06758 PMID: 18344981

41. Mondal AK, Sharma NK, Elbein SC, Das SK (2013) Allelic expression imbalance screening of genes in chromosome 1q21–24 region to identify functional variants for Type 2 diabetes susceptibility. Physiological Genomics 45: 509–520. doi: 10.1152/physiolgenomics.00048.2013 PMID: 23673729

42. Verlaan DJ, Berlivet S, Hunninghake GM, Madore AM, Lariviere M, et al. (2009) Allele-specific chromatin remodeling in the ZPBP2/GSDMB/ORMDL3 locus associated with the risk of asthma and autoimmune disease. American Journal of Human Genetics 85: 377–393. doi: 10.1016/j.ajhg.2009.08.007 PMID: 19732864

43. True HL, Lindquist SL (2000) A yeast prion provides a mechanism for genetic variation and phenotypic diversity. Nature 407: 477–483. PMID: 11028992

44. Gibson G, vanHelden S (1997) Is function of the Drosophila homeotic gene Ultrabithorax canalized? Genetics 147: 1155–1168. PMID: 9383059

45. Richardson JB, Uppendahl LD, Traficante MK, Levy SF, Siegal ML (2013) Histone variant HTZ1 shows extensive epistasis with, but does not increase robustness to, new mutations. PLoS Genetics 9.

46. Suvorov A, Nolte V, Pandey RV, Franssen SU, Futschik A, et al. (2013) Intra-Specific regulatory variation in *Drosophila pseudoobscura*. Plos One 8.

47. Schaefke B, Emerson JJ, Wang TY, Lu MYJ, Hsieh LC, et al. (2013) Inheritance of gene expression level and selective constraints on trans- and cis-regulatory changes in yeast. Molecular Biology and Evolution 30: 2121–2133. doi: 10.1093/molbev/mst114 PMID: 23793114

48. Zhang X, Cal AJ, Borevitz JO (2011) Genetic architecture of regulatory variation in *Arabidopsis thaliana*. Genome Res 21: 725–733. doi: 10.1101/gr.115337.110 PMID: 21467266

49. Wayne ML, Korol A, Mackay TF (2005) Microclinal variation for ovariole number and body size in *Drosophila melanogaster* in 'Evolution Canyon'. Genetica 123: 263–270. PMID: 15954497

50. Cali BM, Anderson P (1998) mRNA surveillance mitigates genetic dominance in *Caenorhabditis elegans*. Mol Gen Genet 260: 176–184. PMID: 9862469

51. Mellert DJ, Truman JW (2012) Transvection is common throughout the Drosophila genome. Genetics 191: 1129–U1130. doi: 10.1534/genetics.112.140475 PMID: 22649078

52. Henikoff S, Comai L (1998) Trans-sensing effects: The ups and downs of being together. Cell 93: 329–332. PMID: 9590167

53. Chen JL, Huisinga KL, Viering MM, Ou SA, Wu CT, et al. (2002) Enhancer action in trans is permitted throughout the Drosophila genome. Proc Natl Acad Sci U S A 99: 3723–3728. PMID: 11904429

54. Johnston RJ, Desplan C (2014) Interchromosomal communication coordinates intrinsically stochastic expression between alleles. Science 343: 661–665. doi: 10.1126/science.1243039 PMID: 24503853

55. Kofler R, Orozco-terWengel P, De Maio N, Pandey RV, Nolte V, et al. (2011) PoPoolation: A toolbox for population genetic analysis of next generation sequencing data from pooled individuals. PLoS One 6.

56. Wu TD, Watanabe CK (2005) GMAP: a genomic mapping and alignment program for mRNA and EST sequences. Bioinformatics 21: 1859–1875. PMID: 15728110

57. Wu TD, Nacu S (2010) Fast and SNP-tolerant detection of complex variants and splicing in short reads. Bioinformatics 26: 873–881. doi: 10.1093/bioinformatics/btq057 PMID: 20147302

58. Pandey RV, Franssen SU, Futschik A, Schlötterer C (2013) Allelic imbalance metre (Allim), a new tool for measuring allele-specific gene expression with RNA-seq data. Molecular Ecology Resources 13: 740–745. doi: 10.1111/1755-0998.12110 PMID: 23615333

59. Marygold SJ, Leyland PC, Seal RL, Goodman JL, Thurmond J, et al. (2013) FlyBase: improvements to the bibliography. Nucleic Acids Research 41: D751–D757. doi: 10.1093/nar/gks1024 PMID: 23125371

60. Graveley BR, Brooks AN, Carlson J, Duff MO, Landolin JM, et al. (2011) The developmental transcriptome of *Drosophila melanogaster*. Nature 471: 473–479. doi: 10.1038/nature09715 PMID: 21179090

61. Robinson MD, Oshlack A (2010) A scaling normalization method for differential expression analysis of RNA-seq data. Genome Biology 11.

62. Robinson MD, McCarthy DJ, Smyth GK (2010) edgeR: a Bioconductor package for differential expression analysis of digital gene expression data. Bioinformatics 26: 139–140. doi: 10.1093/bioinformatics/btp616 PMID: 19910308

63. McCarthy DJ, Chen YS, Smyth GK (2012) Differential expression analysis of multifactor RNA-Seq experiments with respect to biological variation. Nucleic Acids Research 40: 4288–4297. doi: 10.1093/nar/gks042 PMID: 22287627

64. Benjamini Y, Hochberg Y (1995) Controlling the false discovery rate—a practical and powerful approach to multiple testing. Journal of the Royal Statistical Society Series B-Methodological 57: 289–300.

65. Terada A, Okada-Hatakeyama M, Tsuda K, Sese J (2013) Statistical significance of combinatorial regulations. Proc Natl Acad Sci U S A 110: 12996–13001. doi: 10.1073/pnas.1302233110 PMID: 23882073

66. Murali T, Pacifico S, Yu JK, Guest S, Roberts GG, et al. (2011) DroID 2011: a comprehensive, integrated resource for protein, transcription factor, RNA and gene interactions for Drosophila. Nucleic Acids Research 39: D736–D743. doi: 10.1093/nar/gkq1092 PMID: 21036869

67. Prüfer K, Muetzel B, Do HH, Weiss G, Khaitovich P, et al. (2007) FUNC: a package for detecting significant associations between gene sets and ontological annotations. BMC Bioinformatics 8.

68. Luo WJ, Friedman MS, Shedden K, Hankenson KD, Woolf PJ (2009) GAGE: generally applicable gene set enrichment for pathway analysis. BMC Bioinformatics 10.
